# Evolutionary changes in the notochord genetic toolkit: a comparative analysis of notochord genes in the ascidian *Ciona *and the larvacean *Oikopleura*

**DOI:** 10.1186/1471-2148-11-21

**Published:** 2011-01-20

**Authors:** Jamie E Kugler, Pierre Kerner, Jean-Marie Bouquet, Di Jiang, Anna Di Gregorio

**Affiliations:** 1 Department of Cell and Developmental Biology Weill Medical College of Cornell University 1300 York Avenue, Box 60, New York, NY 10065, USA; 2 Sars International Centre for Marine Molecular Biology Thormøhlensgt. 55 N-5008 Bergen, Norway

## Abstract

**Background:**

The notochord is a defining feature of the chordate clade, and invertebrate chordates, such as tunicates, are uniquely suited for studies of this structure. Here we used a well-characterized set of 50 notochord genes known to be targets of the notochord-specific Brachyury transcription factor in one tunicate, *Ciona intestinalis *(Class Ascidiacea), to begin determining whether the same genetic toolkit is employed to build the notochord in another tunicate, *Oikopleura dioica *(Class Larvacea). We identified *Oikopleura *orthologs of the *Ciona *notochord genes, as well as lineage-specific duplicates for which we determined the phylogenetic relationships with related genes from other chordates, and we analyzed their expression patterns in *Oikopleura *embryos.

**Results:**

Of the 50 *Ciona *notochord genes that were used as a reference, only 26 had clearly identifiable orthologs in *Oikopleura*. Two of these conserved genes appeared to have undergone *Oikopleura*- and/or tunicate-specific duplications, and one was present in three copies in *Oikopleura*, thus bringing the number of genes to test to 30. We were able to clone and test 28 of these genes. Thirteen of the 28 *Oikopleura *orthologs of *Ciona *notochord genes showed clear expression in all or in part of the *Oikopleura *notochord, seven were diffusely expressed throughout the tail, six were expressed in tissues other than the notochord, while two probes did not provide a detectable signal at any of the stages analyzed. One of the notochord genes identified, *Oikopleura netrin*, was found to be unevenly expressed in notochord cells, in a pattern reminiscent of that previously observed for one of the *Oikopleura Hox *genes.

**Conclusions:**

A surprisingly high number of *Ciona *notochord genes do not have apparent counterparts in *Oikopleura*, and only a fraction of the evolutionarily conserved genes show clear notochord expression. This suggests that *Ciona *and *Oikopleura*, despite the morphological similarities of their notochords, have developed rather divergent sets of notochord genes after their split from a common tunicate ancestor. This study demonstrates that comparisons between divergent tunicates can lead to insights into the basic complement of genes sufficient for notochord development, and elucidate the constraints that control its composition.

## Background

The notochord is the synapomorphy that gives chordates their name. In vertebrates, this embryonic structure is transient, as it is replaced by the vertebral column, and acts as a powerful organizer, secreting signals required for the patterning of several organs, such as the floor plate, somites, pancreas, heart and dorsal aorta [1]. Developing chordate embryos, including all vertebrates as well as the invertebrate chordates (cephalochordates and tunicates), require a notochord for structural stability, tail elongation, and as an anchor point for muscle contraction [2]. Due to the limited experimental accessibility of the vertebrate notochord and the time and expenses required to generate transgenic vertebrate embryos, a considerable amount of the recent research on notochord genes and their transcriptional regulation has been carried out in invertebrate chordates, particularly ascidians, such as *Ciona intestinalis *[3-6] and *Halocynthia roretzi *[7,8], which together with thaliaceans and larvaceans [9] are part of the tunicate subphylum. Studies in *Ciona intestinalis *have identified numerous notochord genes, both evolutionarily conserved [10-13] and lineage-specific [14]. Parallel studies of a subset of orthologs of *Ciona *notochord genes have shown their expression in the mouse notochord [15].

While these observations indicate that ascidians represent a valid model for studying the evolutionary origins of the notochord, they also raise questions on the applicability of these findings to other invertebrate chordates. In fact, a considerable degree of variability in the structure and genetic makeup of the notochord has been found among invertebrate chordates; the most striking example of such variability is arguably the notochord of amphioxus (cephalochordate), which is functionally related to the ascidian notochord but expresses numerous muscle genes [16,17], in addition to typical notochord markers [18,19]. Even within ascidians, divergent species such as *Ciona *(Order Enterogona) and *Halocynthia *(Order Pleurogona), employ different molecular strategies to build morphologically similar structures, as shown by studies on the molecular control of secondary notochord induction [20,21].

In this study, we have begun probing the extent of variation tolerable in the notochord gene complement used by representatives of two classes of tunicates. To this aim, we tested in the larvacean *Oikopleura dioica *the expression patterns of notochord genes previously characterized in the ascidian *Ciona intestinalis*.

**Figure 1:**
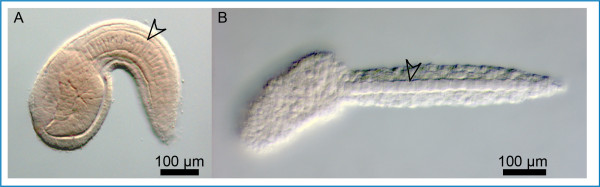
**The notochord of *Ciona intestinalis *and *Oikopleura dioica *embryos**. Microphotographs of a *Ciona intestinalis *embryo (A) and an *Oikopleura dioica *embryo (B) at comparable developmental stages. In each panel the notochord is highlighted by an arrowhead. Anterior is to the left and dorsal is up.

*Oikopleura dioica *(hereinafter *Oikopleura*) is a small pelagic tunicate which possesses the most compact genome identified in any chordate, at only 72 Mb [22]. *Oikopleura *has a 6-day life cycle at 15°C [23] and can be cultured *in vitro *in a suitably staffed laboratory. *Oikopleura *and other larvaceans are unique among tunicates in that they retain the notochord throughout their lifespan [9,24]. Differently from the notochord of a *Ciona *tailbud, which consists of 40 cells and is flanked on each side by 18 muscle cells [25], the notochord of a tailbud *Oikopleura *embryo contains only 20 cells (19 columnar cells plus one round terminal cell), flanked on each side by 10 muscle cells [9]. While in *Ciona *the 40 notochord cells cease to divide by the early tailbud stage [25] and are gradually reabsorbed at metamorphosis [26], the notochord cells in *Oikopleura *increase their number through additional mitoses, which bring their final count to 120-160 by the third day of life [27]. Nevertheless, the overall "stack-of-coins" arrangement of the notochord cells and the morphogenetic events that characterize notochord differentiation, including the formation of vacuoles which eventually coalesce to form a hollow lumen, appear quite similar between these tunicates [27] (Figure. 1). The main difference identified so far at the molecular level is in the expression of *Hox *genes, which are absent from the *Ciona *notochord [28] but are expressed in the *Oikopleura *notochord in a spatial sequence resembling the colinear expression seen in vertebrates [29]. Most importantly, the *Oikopleura *notochord also expresses Brachyury [30], a T-box transcription factor necessary for notochord formation in *Ciona *and in all chordates analyzed thus far [31,32]. Most of the currently known Brachyury-downstream notochord genes have been identified in *Ciona intestinalis *[13,33,34].

The conserved notochord expression of *Brachyury *homologs in *Ciona *and *Oikopleura *prompted us to use the 50 notochord genes identified so far as *bona fide Ciona *Brachyury (Ci-Bra) targets to assess whether, and to what extent, *Oikopleura *retains the notochord gene battery identified in *Ciona*. The work presented here therefore begins by surveying the *Oikopleura *genome and by identifying *in silico *orthologs of Ci-Bra target genes. The expression of these genes was then analyzed by WMISH on *Oikopleura *embryos fixed at embryonic stages that correspond, approximately, to the stages at which notochord expression is detected in *Ciona *[27] as well as on embryos at later developmental stages. Our results show that of the 50 Ci-Bra target genes that were used as a starting point, 24 did not have detectable counterparts in the *Oikopleura *genome. We were able to clone and analyze 28 of the remaining 30 genes (26+4 lineage-specific duplicate genes), and found that 13 of these genes were expressed in the notochord, seven were expressed at low levels throughout the tail, thus possibly including the notochord, six were clearly expressed in tissues other than the notochord, and two were not detectable under the experimental conditions employed. Taken together, our results suggest a considerable divergence in the notochord genetic toolkit between *Ciona *and *Oikopleura *and begin to pinpoint genes and pathways that might be conserved in this structure as a result of evolutionary constraints.

## Results and Discussion

### Lineage-specific duplication events among *Oikopleura *orthologs of *Ciona *notochord genes

To identify notochord genes in *Oikopleura*, we used as a starting point a list of 50 notochord genes which had been previously characterized in *Ciona intestinalis *as Ci-Bra transcriptional targets [13,33] (Additional file 1 and references therein). One gene, *Ci-collagen α1 type XVIII 1*, included in a previously published list of Ci-Bra targets [33], was excluded from this study due to discrepancies in gene model references. We used the reciprocal best BLAST hit method [35,36] to detect and characterize *bona fide *orthologs of Ci-Bra targets present in the *Oikopleura *genome. Briefly, we used both the allelic and reference first draft genome assembly of *Oikopleura*, as well as 18,020 computer-generated gene models (http://www.genoscope.cns.fr/externe/GenomeBrowser/Oikopleura/ and [37]) as databases for our BLASTX or TBLASTN queries [38], using *Ciona *JGI version 1.0 translated gene models (Additional file 1) for first-pass screens and subsequently comparing the results with the updated *Ciona *KH (Kyoto Hoya) gene models [39] as reported in the ANISEED database (http://www.aniseed.cnrs.fr/). Out of 50 queries, only 26 yielded identifiable positive reciprocal best BLAST hits (Additional file 1).

**Figure 2:**
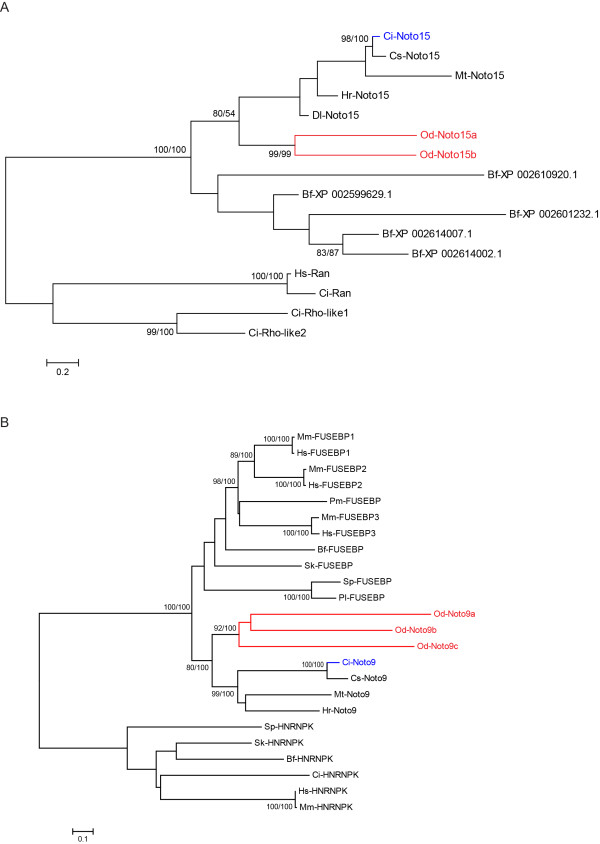
**Phylogenetic analyses of *Noto15 *and *Noto9 *duplicates found in *Oikopleura***. Phylogenetic reconstructions performed for (A) *Noto15 *and (B) *Noto9 *(Far UpStream Element Binding Proteins, or FUSEBPs) duplicates found in *Oikopleura*. Trees were obtained from neighbor-joining analyses and rooted using heterogeneous nuclear ribonucleoprotein K (HNRNPK) and Rho-like and Ran protein sequences, respectively, as outgroups. Statistical support values ≥80% obtained with different methods are included over conserved nodes; the first number indicates the bootstrap support in neighbor-joining analysis (1000 bootstrap replicates), the second number reports the posterior probabilities in Bayesian inference analysis. Note that a single value of 54% posterior probability was kept on the node grouping all tunicate *Noto15 *orthologs. Abbreviations: Ci: *Ciona intestinalis*; Cs: *Ciona savignyi*; Mt: *Molgula tectiformis*; Hr: *Halocynthia roretzi*; Dl: *Diplosoma listerianum*; Od: *Oikopleura dioica*; Bf: *Branchiostoma floridae*; Hs: *Homo sapiens*; Mm: *Mus musculus*; Pm: *Petromyzon marinus*; Sk: *Saccoglossus kowalevskii*; Sp: *Strongylocentrotus purpuratus*; Pl: *Paracentrotus lividus*. The scale bars indicate the branch length that corresponds to the number of substitutions per residue.

**Figure 3:**
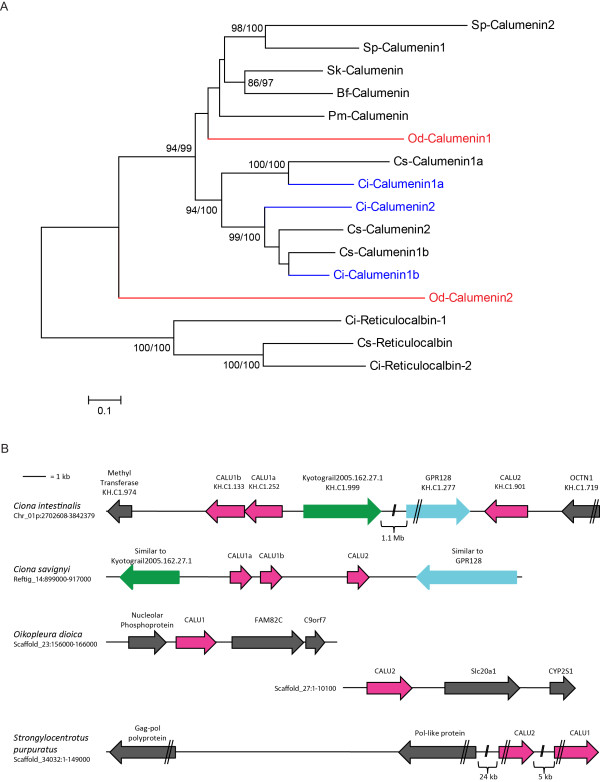
**Phylogenetic analyses and genomic organization of *Calumenin *orthologs found in deuterostome genomes**. (A) Phylogeny of the predicted proteins encoded by the duplicate *Calumenin *genes found in *Oikopleura*. The tree was constructed based upon neighbor-joining analyses and rooted using Reticulocalbin. Statistical support values ≥80% obtained with different methods are included over conserved nodes, as described in Figure. 2. Abbreviations are as in Figure. 2. (B) Schematic depictions of the genomic regions encompassing *Calumenin *paralogs in *Ciona intestinalis*, *Ciona savignyi*, *Oikopleura dioica *and *Strongylocentrotus purpuratus*. The names of the genes flanking the *Calumenin *genes are either those indicated in the genome browsers of the respective species or those identified using the reciprocal best BLAST hit method.

For each reciprocal best BLAST hit, the corresponding *Oikopleura *genomic region, gene model and available ESTs sequences were collected and aligned to allow manual curation prior to cloning (data not shown). This procedure uncovered possible duplication events. In particular, we noticed that two different genes seemed to have been duplicated in *Oikopleura*, *Od-Noto15 *and *Od-Calumenin*, while a third one, *Od-Noto9*, was found to be present in three copies. For this reason, in the case of these genes (Figures. 2, 3 and Additional file 2) and a few more (data not shown), we resorted to the generation of phylogenetic reconstructions in order to minimize mis-assignments of orthologous relationships.

To construct a phylogenetic tree of orthologs of *Ci-Noto15 *(Figure. 2A), we performed a BLAST search against GenBank (http://www.ncbi.nlm.nih.gov/genbank/). Interestingly, this search retrieved predominantly tunicate sequences, from the ascidians *Ciona savignyi*, *Halocynthia roretzi*, *Molgula tectiformis *and *Diplosoma listerianum*, as opposed to other deuterostome sequences. Extensive BLAST searches of specific databases of genomic and EST sequences, including *Strongylocentrotus purpuratus*, *Saccoglossus kowalevskii*, *Branchiostoma floridae*, *Petromyzon marinus *and *Mus musculus*, yielded hits with poor e-values (below e^-12^), even though a conserved Ras-like domain [40] was detected in the Ci-Noto15 protein sequence. Among those hits, five uncharacterized predicted protein sequences from *Branchiostoma floridae *(amphioxus) with e-values between 2e^-4 ^and 6e^-12 ^were retrieved (Figure. 2A). To root the tree, we used two different representative sequences of the Ras-superfamily (Ran- and Rho-like) as outgroups. Surprisingly, tunicate *Noto15 *putative orthologs bundled together, while no clear relationships were found with any of the five predicted amphioxus proteins, which group themselves separately in our tree (Figure. 2A), though without sufficient statistical support. This suggests that we retrieved *bona fide *tunicate *Noto15 *orthologs which belong to either a novel family specific to the tunicate lineage, or to a group of divergent members eluding identification, or that the *Noto15 *and unidentified amphioxus genes represent a group of chordate-specific genes that arose early during the evolutionary history of the lineage, and has been preserved in cephalochordates and tunicates but secondarily lost in the vertebrate lineage. Moreover, the two putative *Noto15 *orthologs found in *Oikopleura *are grouped together with a statistical support of 99% by both neighbor-joining and Bayesian inference methods; this suggests that these copies arose from a duplication event specific to *Oikopleura*. In an effort to trace back this event, we studied the genomic localization and neighboring genes of *Od-Noto15a *and *Od-Noto15b*, but we did not find conclusive syntenic relationships between these genes, either intra- or interspecifically (data not shown).

### Three copies of the *Ciona Noto9/FUSEBP *gene are present in the *Oikopleura *genome

We used the reciprocal best BLAST hit method to identify orthologs of *Ci-Noto9 *in other deuterostome sequence databases. All best hits corresponded to Far UpStream Element Binding Proteins (FUSEBPs) [41] and were found in diverse animals spanning the main branches of the deuterostome lineage (Figure. 2B). FUSEBPs belong to the large superfamily of RNA-binding proteins, and more specifically to the K-homology (KH) RNA-binding domain [42]. We therefore rooted the tree with various deuterostome heterogeneous nuclear ribonucleoprotein K (HNRNPK) protein sequences as outgroups. In most deuterostomes, a single copy of FUSEBP (*Noto9*) was found, while 3 FUSEBPs were found in mammalian genomes as well as in *Oikopleura*. However, our phylogenetic analysis suggests that the three copies found in mammals and *Oikopleura *arose from independent lineage-specific duplication events. In fact, vertebrate FUSEBPs group together with a statistical support of 98% with the neighbor-joining method and 100% of posterior probabilities as calculated by the Bayesian inference method, whereas Od-Noto9a, Od-Noto9b and Od-Noto9c group together with a statistical support of 92% and 100%, respectively, in a related yet separate tunicate monophyletic group. Despite our efforts, no other copies were found in other tunicate databases, which tentatively indicates that these duplications were isolated events circumscribed to the peculiar genomic evolutionary history of *Oikopleura*; however, this conclusion might have to be revisited once complete genomic sequences become available for other tunicates.

### Gene duplications, synteny and genomic rearrangements: the case of the *Calumenin *genes

Another phylogenetic tree was constructed to study the evolutionary history and reciprocal relationships of *Calumenin *genes from basal deuterostomes (Figure. 3A). Calumenin proteins belong to a family of low-affinity Ca^2+^-binding, multiple EF-hand proteins which includes Cab45, Reticulocalbin, ERC-55, and Calumenin (CREC) [43]. As numerous copies of these genes are present in mammalian genomes, and as these were not annotated following a rigorous phylogenetic reconstruction, we restricted our analysis to basal deuterostomes by excluding gnathostome sequences from this study. To root the tree we employed ascidian Reticulocalbin sequences. We found multiple copies of *Calumenin *genes in invertebrate deuterostome genomes: two copies in the sea urchin *Strongylocentrotus purpuratus*, two in *Oikopleura *and three copies in the sister species *Ciona intestinalis *and *Ciona savignyi*. Despite our extensive efforts, only single-copy genes were found in amphioxus, enteropneust and lamprey genomes. We also observed that in our phylogenetic reconstructions putative *Calumenin *orthologs in *Oikopleura *were not satisfactorily linked to any monophyletic group in this tree. Although Od-Calumenin1 is found inside a statistically supported group with all other Calumenin sequences, Od-Calumenin2 is not reliably linked to this group, likely because of the high evolutionary rate of divergence of *Oikopleura *genes. In addition, the two copies found in the sea urchin genome seem to have arisen from a duplication event independent from that which gave rise to the three copies found in ascidians. In order to trace these duplication events, we analyzed the genomic location and flanking genes of *Calumenin *paralogs found in *Ciona*, *Strongylocentrotus *and *Oikopleura *(Figure. 3B). This allowed us to gain more insights into the evolutionary history of these genes in different genomes, as we found that all *Calumenin *paralogs map to a single chromosome (or sequence scaffold) in ascidians as well as in the sea urchin. In particular, the *Calumenin *paralogs that we found in *Ciona savignyi *are clustered in a contiguous arrangement on Reftig14, which suggests that they originated from two tandem duplication events. In *Ciona intestinalis*, the three *Calumenin *genes are found on the same chromosome, but *Calumenin1a *and *Calumenin1b *are separated by more than 1.1 Mb from *Calumenin2 *(Figure. 3B). Nevertheless, we found that *Calumenin *paralogs are flanked in both *Ciona *species by two genes: a gene similar to the unannotated gene model Kyotograil2005.162.27.1 (green arrows in Figure. 3B), and a gene annotated as GPR128 (G-protein coupled receptor 128 precursor) (light blue arrows in Figure. 3B). These observations indicate that the linked arrangement found in *Ciona savignyi *might have been split by a chromosomal rearrangement in *Ciona intestinalis*. The fact that the genes flanking *Calumenin *paralogs are identical between the two *Ciona *species but different from those found in the sea urchin, strengthens the hypothesis that the echinoderm *Calumenin *paralogs arose from distinct duplication events. In *Oikopleura*, *Calumenin *paralogs map to different scaffolds and are flanked by genes that are not found in the vicinity of the ascidian *Calumenin *paralogs, which limits the conclusions that can be drawn on their origin.

### Identification of novel *Oikopleura *notochord genes

**Figure 4:**
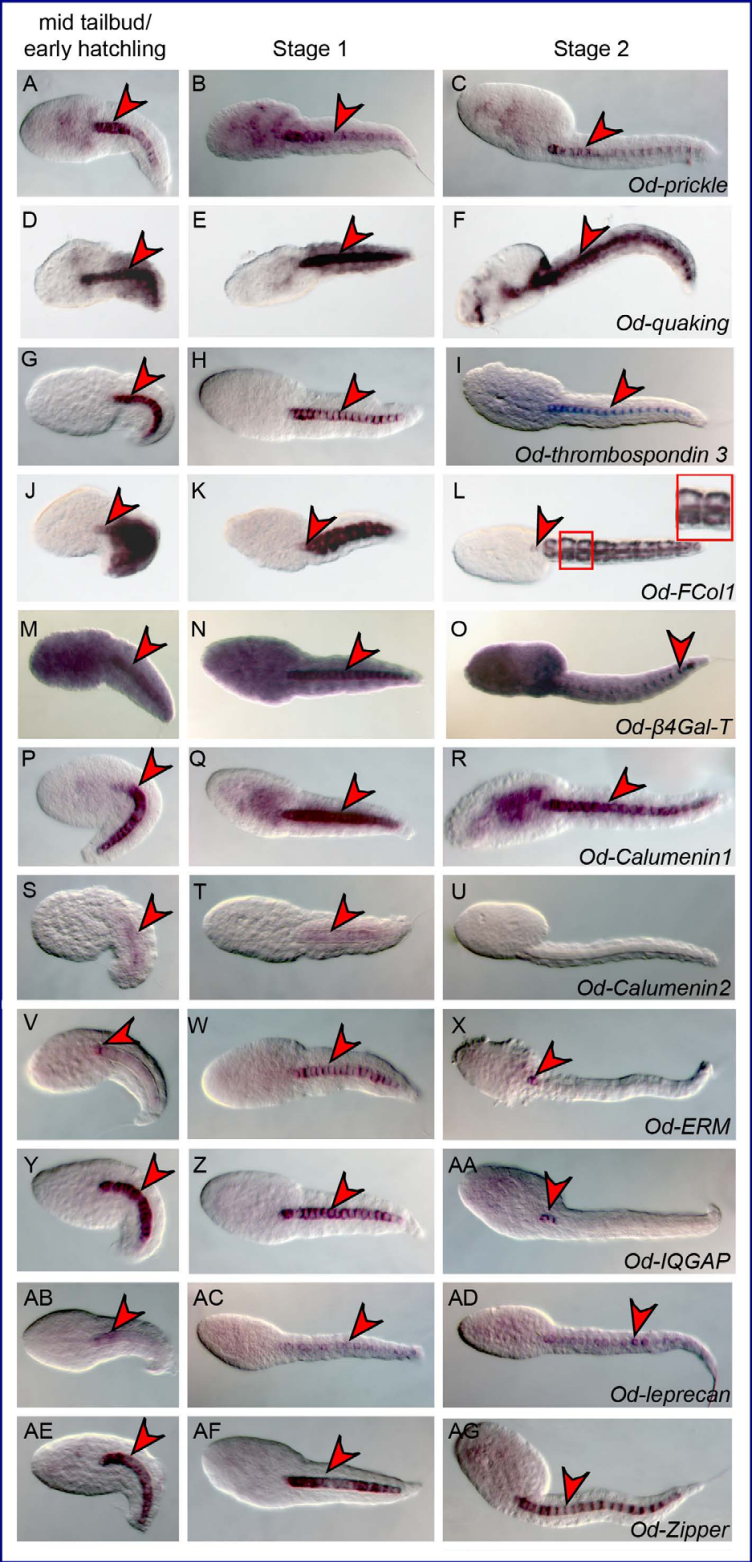
**Identification of novel notochord genes in *Oikopleura***. (A-AG) Microphotographs of *Oikopleura dioica *embryos hybridized *in situ *with antisense probes against the genes indicated at the bottom right of each row. The developmental stages tested are indicated in the column headings. Embryos are oriented with their anterior ends to the left and their dorsal sides up. Red arrowheads highlight notochord staining. The inset in (L) shows stained muscle cells.

**Figure 5:**
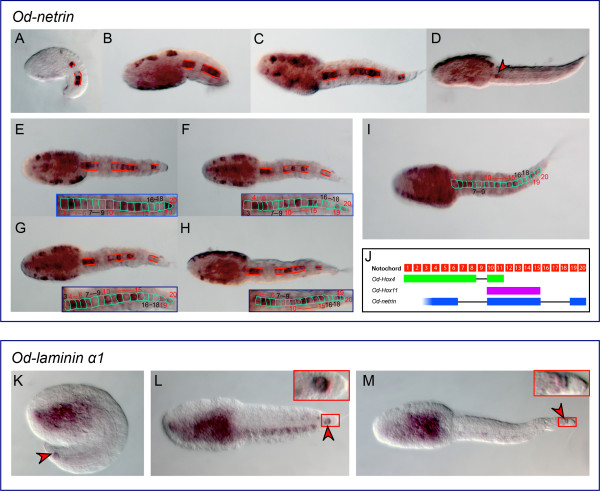
**Uneven expression of *Oikopleura netrin *and *laminin α1 *in the notochord**. (A-I) Microphotographs of *Oikopleura *embryos at the mid-tailbud/early hatchling (A), late tailbud (B), Stage 1 (C,E-I), and Stage 2 (D), hybridized *in situ *with a digoxigenin-labeled *Od-netrin *antisense probe. All embryos are oriented with anterior to the left. Red lines delineate the notochord domains of *Od-netrin *expression. Individual notochord cells are outlined in aqua in (I) and in the insets showing the magnified notochord (E-H). In (D), a red arrowhead indicates residual staining in one of the notochord cells close to the trunk-tail boundary. (J) Schematic representation of the *Oikopleura *notochord cells (20 numbered red squares), followed, for comparison purposes, by a delineation of the *Od-Hox4 *(green), *Od-Hox11 *(purple) [29], and *Od-netrin *(blue) expression patterns. (K-M) Microphotographs of *Oikopleura *embryos at the mid-tailbud/early hatchling (K), Stage 1 (L), and Stage 2 (M), hybridized *in situ *with a digoxigenin-labeled *Od-laminin α1 *antisense probe. A red arrowhead indicates the staining in the terminal notochord cell, which is magnified in the insets in panels (L) and (M).

**Figure 6:**
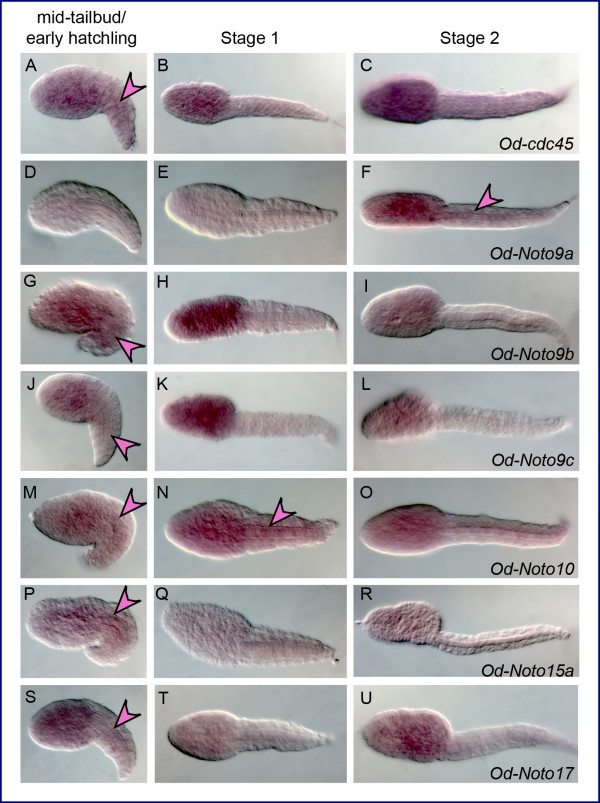
***Oikopleura *genes expressed throughout the tail**. (A-U) *Oikopleura *embryos hybridized *in situ *with antisense probes for the genes indicated at the bottom right of each row. The developmental stages are indicated in the column headings; all embryos are oriented with anterior to the left and dorsal up, with the exception of the embryos in panels (N) and (T), which are shown as dorsal views. Pink arrowheads indicate the notochord in all embryos showing diffuse expression throughout the tail.

**Figure 7:**
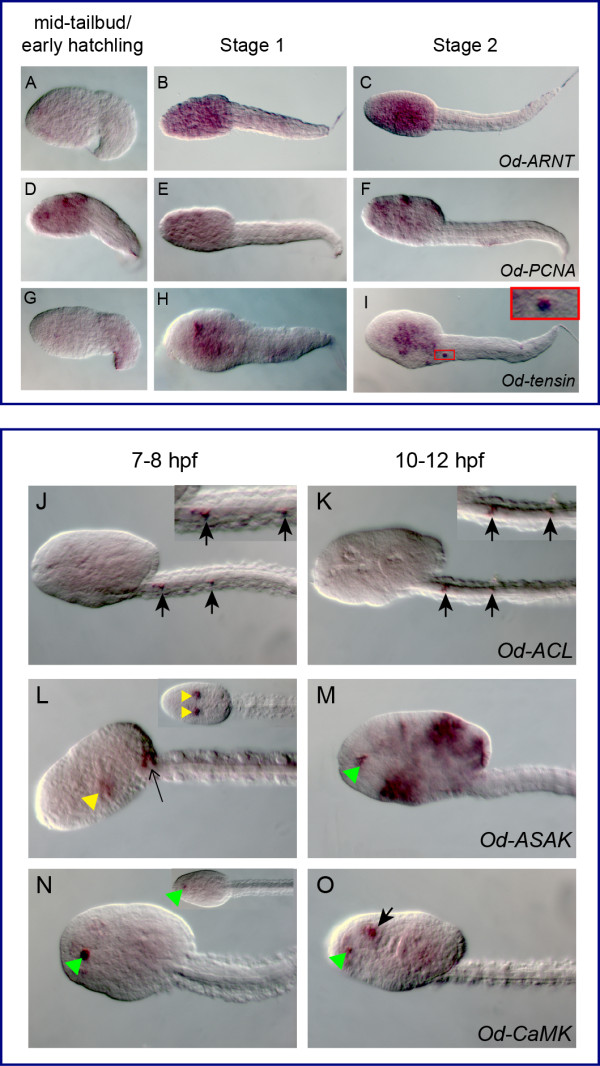
***Oikopleura *orthologs of *Ciona *notochord genes expressed in tissues other than the notochord**. (A-I) *Oikopleura *embryos hybridized *in situ *with the antisense probes indicated at the bottom right of each row. The developmental stages are indicated in the column headings; all embryos are oriented with anterior to the left and dorsal up. The inset in (I) shows a magnified view of a cell specifically stained by *Od-tensin*, likely part of the caudal ganglion. (J-O) High-magnification views of *Oikopleura *embryos at 7-8 and 10-12 hpf, hybridized *in situ *with antisense probes for *Od-ACL *(J,K), *Od-ASAK *(L,M) and *Od-CaMK *(N,O). Black arrows in (J,K) indicate tail neurons (magnified in the insets); yellow arrowheads indicate spiracles; a thin black arrow in (L) indicates putative cells of the base of the Langerhans receptor; green arrowheads indicate the ciliary funnel (M-O) and a black arrow in (O) indicates signal detected in the cerebral ganglion, at the level of the statocyst.

Specific cDNA fragments corresponding to 28 *Oikopleura *genes identified as *bona fide *orthologs of *Ciona *notochord genes were cloned by PCR (Additional file 3), and used to synthesize antisense RNA probes that were tested on *Oikopleura *embryos by WMISH. Time-points of 2.5 (mid-tailbud/early hatchling), 4 (Stage 1), and 5.5 (Stage 2) hpf at 21°C were used, as these corresponded, approximately, to the developmental stages at which the *Ciona *counterparts are known to be expressed [27]. Of the 28 probes tested, two (*Od-Noto15b *and *Od-pellino*) yielded no signal at any of the stages analyzed, despite repeated attempts. Thirteen of the genes tested were expressed in all or part of the notochord in at least one developmental stage: *Od-prickle*, *Od-quaking, Od-thrombospondin 3*, *Od-Fibrillar collagen 1 *(*Od-FCol1*)*, Od- β1,4-Galactosyltransferase *(*Od-β4-GalT*), *Od-Calumenin1*, *Od-Calumenin2*, *Od-Ezrin/radixin/moesin *(*Od-ERM*), *Od-IQ motif-containing GTPase-activating protein *(*Od-IQGAP*), *Od-leprecan*, *Od-Zipper *(Figure. 4 and Additional file 4), *Od-netrin *and *Od-laminin α1 *(Figure. 5 and Additional file 4). Seven probes, *Od-cdc45, Od-Noto9a*, *Od-Noto9b*, *Od-Noto9c*, *Od-Noto10*, *Od-Noto15a *and *Od-Noto17*, yielded a diffuse, weak signal in the tail, which might indicate low expression levels in the notochord (Figure. 6 and Additional file 4). Finally, six of the probes tested revealed distinct staining patterns which did not include notochord: *Od-aryl hydrocarbon receptor nuclear translocator *(*Od-ARNT*), *Od-proliferating cell nuclear antigen *(*Od-PCNA*), *Od-tensin*, *Od-ATP citrate lyase *(*Od-ACL*), *ATP sulfurylase/adenosine 5*'*-phosphosulfate (APS) kinase (Od-ASAK) *and *Od-calmodulin-dependent protein kinase *(*Od-CaMK*) (Figure. 7 and Additional file 4).

We found that conserved *Oikopleura *notochord genes exhibited variable expression patterns (Figure. 4), and some of these genes were expressed in other tissues in addition to the notochord. *Od-prickle *(Figure. 4A-C) and *Od-Calumenin1 *(Figure. 4P-R) were also expressed in some areas of the trunk, while *Od-quaking *(Figure. 4D-F), *Od-FCol1 *(Figure. 4J-L), and possibly *Od-β4-GalT *(Figure. 4M-O) were expressed in muscle cells as well as in notochord cells. Interestingly, similarly to *Od-quaking *and *Od-FCol1*, *Ci-quaking *and *CiFCol1 *are also expressed in notochord and muscle cells, with the latter gene being expressed also in nervous system and endoderm in addition to these territories [13,44]. *Od-thrombospondin 3 *(Figure. 4G-I), *Od-Calumenin2 *(Figure. 4S-U), *Od-ERM *(Figure. 4V-X), *Od-IQGAP *(Figure. 4Y-AA), *Od-leprecan *(Figure. 4AB-AD) and *Od-Zipper *(Figure. 4AE-AG) were notochord-specific at these stages. Additional variability was seen in the developmental windows of expression of these genes. While most genes were expressed in the notochord at all time-points between mid-tailbud/early hatchling and Stage 2, four of them, *Od-β4-GalT *(Figure. 4M-O), *Od-Calumenin2 *(Figure. 4S-U), *Od-ERM *(Figure. 4V-X) and *Od-IQGAP *(Figure. 4Y-AA), were expressed only transiently and their expression had more or less faded by Stage 2. For eight of these *Oikopleura *notochord genes we identified expression in notochord precursors at the neurula stage (Additional file 5, panels A-H); remarkably, however, none of the *Oikopleura *genes were expressed in the notochord after Stage 2 (Additional file 6 and data not shown). These observations confirm the correlation previously described between the morphogenetic events of notochord formation for *Ciona intestinalis *and *Oikopleura dioica *[45], according to which by eight hours after fertilization *Oikopleura dioica *embryos reach the ascidian stage IV, which is characterized by completion of vacuolization and formation of a hollow tubular notochord.

While little is known about the possible role of the putative non-muscle myosin heavy chain encoded by *Zipper *in notochord formation, the conserved Ci-Bra target orthologs expressed in the *Oikopleura *notochord encode proteins that in other organisms have been reported to serve a wide range of functions. *Od-FCol1*, *Od-thrombospondin 3 *and *Od-leprecan *encode components of the extracellular matrix which are required for notochord integrity [15,46,47]. *prickle*, which encodes a planar cell polarity protein, has been shown to be necessary for notochord cell intercalation in both *Ciona intestinalis *and *Ciona savignyi *[48,49]. Ezrin-radixin-moesin, which belongs to a class of proteins which help to organize the cell cortex through their cytoskeletal and transmembrane interaction domains [50], has been shown to be required for notochord cell elongation in *Ciona intestinalis *[49]. The conserved notochord genes, *quaking*, *β4-GalT*, *Calumenin1*, *Calumenin2 *and *IQGAP*, which encode, respectively, an RNA-binding protein, an enzyme involved in lactose biosynthesis, Ca^2+^-binding proteins, and a scaffolding protein of the Rho family, respectively, have also been found to be expressed in the notochord of other chordates [51,52]. In particular, the Quaking RNA-binding protein has been shown to be necessary for notochord formation in *Xenopus*, where it stabilizes the mRNAs for proteins that are required for notochord development, including that of *Xenopus Brachyury *[51], while XIQGAP1 has been reported to be predominantly localized to lamellipodia and filopodia in the *Xenopus *notochord, at the time when the morphogenetic movements leading to notochord formation take place [52].

### *Oikopleura netrin*, *laminin α1*, and the molecular heterogeneity of the notochord of basal chordates

Among the *Oikopleura *genes for which we found conserved notochord expression, one, *Od-netrin*, displayed a strikingly uneven pattern, being detectable only in a subset of notochord cells. Proteins of the Netrin family are diffusible chemoattractants required for axon guidance in a wide variety of animals [53]. *Od-netrin *was first detected in a few notochord precursors at the neurula stage (Additional file 5, panel H); at the mid-tailbud/early hatchling stage and Stage 1, notochord expression was distinctively discontinuous (Figure. 5A-C) and the signal began fading from the notochord cells shortly after, by Stage 2, when it was detected only in anterior notochord cell(s) (Figure. 5D and data not shown), although it remained clearly visible in the trunk. While in mid-tailbud embryos there were two groups of stained notochord cells separated by a gap, in all embryos at Stage 1 we could distinguish three groups of contiguous stained cells separated by two gaps of blank cells (insets in Figure. 5E-H; Figure. 5I). The anterior-most segment of stained notochord contained on average 2-3 cells, while the middle segment contained five cells, displaying variable levels of staining (insets in Figure. 5F,G) and the posterior-most segment contained two cells. The gaps usually spanned three cells (insets in Figure. 5E-H; Figure. 5I). The pattern that we observed for *Od-netrin *is substantially different from that reported for *Ci-netrin*, as the latter is expressed quite homogeneously in all notochord cells at all stages analyzed [54].

A discontinuous pattern similar to the pattern that we observed for *Od-netrin *had been previously reported for one of the *Oikopleura Hox *genes, *Od-Hox4*, which shows a distinct gap between two stained regions of the notochord [29] (schematic in Figure. 5J); another *Oikopleura Hox *gene, *Od-Hox11*, is expressed only in a stretch of six contiguous notochord cells ([29]; Figure. 5J). However, the *Od-netrin *pattern cannot be perfectly superimposed to the pattern seen for either of the two *Od-Hox *genes, suggesting that other transcription factors and/or additional regulatory mechanisms might be modulating this peculiar expression. Within tunicates, a similarly irregular pattern in notochord cells has been reported for *Ciona multidom*, a Ci-Bra-downstream notochord gene that does not have counterparts in *Oikopleura *(Additional file 1). However, *Ci-multidom *is expressed in a completely mosaic fashion, whereby expression surfaces randomly in a variable number of notochord cells whose position along the anterior-posterior axis of the notochord is randomized as well [55]. Remarkably, an amphioxus *netrin *gene, *AmphiNetrin*, has been described to be expressed with variable intensity along the notochord in a stage-dependent fashion [56]. In particular, at neurulation, *AmphiNetrin *is strongly expressed in the anterior extension of the notochord underlying the cerebral vesicle, while in early larvae expression in this region is no longer detectable [56].

The functional meaning and developmental consequences of a discontinuous source of Netrin in the notochord remain to be elucidated. However, it is intriguing that the *Oikopleura *caudal nerve cord contains ganglia scattered along its length at irregular intervals [45], from which thin peripheral nerve fibers project ventrally on the surface of the notochord cells [57]. In hatched larvae around 4 hpf (at 21°C), which is approximately the stage shown in Figure. 5C and 5E-I, the first nerve fibers become visible in the caudal ganglion and in the nerve cord, dorsal to the notochord [58], and by 4 hours and 45 minutes after fertilization the full complement of post-mitotic neurons has already been reached [45]. It is therefore conceivable that the unevenly distributed sources of Netrin that we have identified in the notochord might serve a specific role in the guidance of the peripheral nerve fibers which emanate from the ganglia of the nerve cord.

Another peculiar expression pattern has been revealed by this study for *Od-laminin α1*. In embryos at the mid-tailbud stage, this gene is visibly expressed in the trunk and in what appears to be the posterior-most of the 20 notochord cells present at this stage (Figure. 5K). This notochord cell is also known as the "terminal" cell or "t-cell" [9,27] and displays a characteristic round shape (insets in Figure. 5L-M). Transient staining was also observed in other cells of the tail flanking the notochord (Figure. 5L); however, no signal was detected in notochord cells other than the terminal cell at either earlier (data not shown) or later stages (Figure. 5K-M, Additional file 6, panels A,B and data not shown). In embryos at 7-8 hpf and 10-12 hpf, expression of *Od-laminin α1 *is detected in a small region of the developing intestine and also in the subchordal cells [9], whose function is yet to be elucidated (Additional file 6, panels A,B and insets therein).

### *Oikopleura *genes expressed at low levels throughout the tail

Seven of the *Oikopleura *probes employed in this study yielded a more or less diffuse staining throughout the tail at one or more of the developmental stages that were tested (Figure. 6). The diffuse staining that we observed in the tail in these cases appeared to encompass the notochord (pink arrowheads in Figure. 6A,F,G,J,M,N,P,S) and was frequently accompanied by staining in the trunk. In the majority of cases, the diffuse staining in the tail was observed exclusively at the mid-tailbud/early hatchling stage (Figure. 6A,G,J,P,S). However, in the case of *Od-Noto9a*, only embryos at Stage 2 displayed a weak signal in some regions of the notochord (Figure. 6F). Interestingly, this diffuse staining was observed for all three *Noto9 *genes (Figure. 6D-L), although while the spatio-temporal expression pattern of *Od-Noto9b *and *Od-Noto9c *appear practically identical, expression of *Od-Noto9a *becomes detectable only at Stage 2. In embryos at 7-8 hpf, expression of all three genes became confined to the pharynx, in a region likely including the developing ciliary funnel (Additional file 6, panels C,E,G). In embryos at 10-12 hpf, expression in the presumptive ciliary funnel was still detectable for *Od-Noto9b *and *Od-Noto9c *(Additional file 6, panels F,H) but not for *Od-Noto9a *(Additional file 6, panel D).

### *Oikopleura *genes that are not expressed in notochord cells

Six of the *Oikopleura *orthologs of *Ciona *notochord genes analyzed in this study turned out to be expressed in tissues other than the notochord (Figure. 7). Three of these genes, *Od-ARNT *(Figure. 7A-C), *Od-PCNA *(Figure. 7D-F) and *Od-tensin *(Figure. 7G-I) were predominantly expressed in the trunk. *Od-tensin *was also transiently expressed in what might be one of the neurons of the caudal ganglion (inset in Figure. 7I). For three additional genes, *Od-ACL*, *Od-ASAK *and *Od-CaMK*, we could not detect any signal in neurulae, mid-tailbud/early hatchlings and Stages 1 and 2 (data not shown); however, we were able to detect convincing staining in embryos at later stages, namely 7-8 hpf and 10-12 hpf (Figure. 7J-O). In particular, *Od-ACL *was detected in a subpopulation of tail neurons, some of which grouped in the caudal ganglion, at both these stages (Figure. 7J,K). *Od-ASAK *expression at 7-8 hpf was localized to the spiracles and to some of the posterior trunk cells that eventually constitute the base of the Langerhans receptors (Figure. 7L and data not shown), and to the presumptive ciliary funnel and various epithelial regions at 10-12 hpf (Figure. 7M). The *Od-CaMK *probe labeled the ciliary funnel at both 7-8 hpf and 10-12 hpf (Figure. 7N,O); in addition, at 10-12 hpf, a strong signal was detected in the cerebral ganglion, in an area that seems to encompass the statocyst (Figure. 7O).

**Figure 8:**
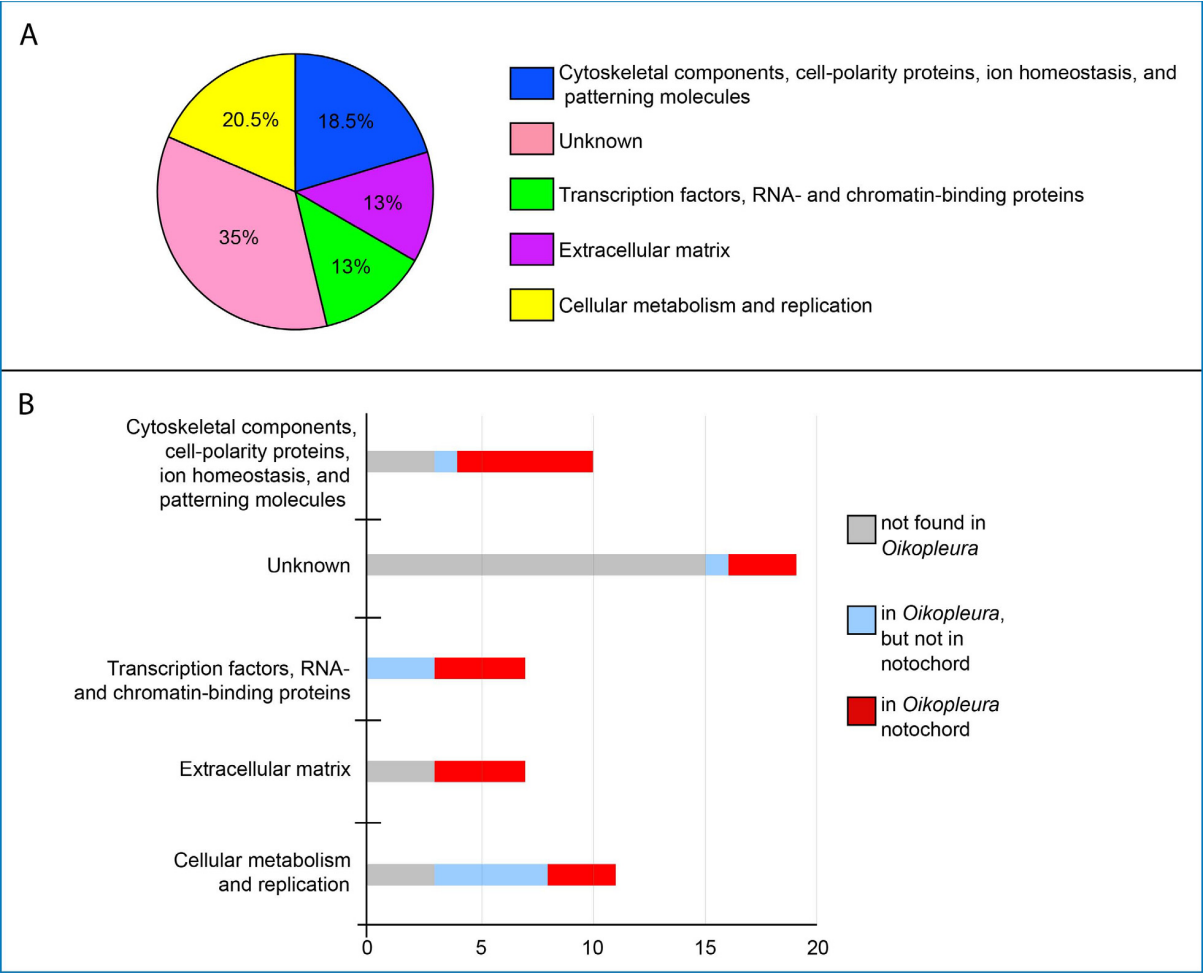
**Conservation of *Ciona *notochord genes in *Oikopleura***. (A) Graph showing the distribution of Ci-Bra target genes based on their putative function. Putative functions were ascertained from the published literature [13,35]. (B) Graph showing the conservation of Ci-Bra target genes in *Oikopleura*. The *x*-axis indicates the number of genes in each category. **Figure S1. Sequence alignments used for phylogenetic reconstructions**. Multiple alignments of the Noto15, Noto9 and Calumenin protein sequences used to construct the phylogenetic trees shown in Figures. 2 and 3. Shaded areas match a cutoff threshold of 80% identity/similarity calculated according to the BLOSUM62 similarity scoring matrix [76]. Abbreviations: Hs: *Homo sapiens*; Ci: *Ciona intestinalis*; Bf: *Branchiostoma floridae*; Cs: *Ciona savignyi*; Hr: *Halocynthia roretzi*; Mt: *Molgula tectiformis*; Dl: *Diplosoma listerianum*; Od: *Oikopleura dioica*. **Figure S2. Whole-mount *in situ *hybridization of *Oikopleura *neurulae**. *Oikopleura *embryos at the neurula stage (1.8 hpf), hybridized *in situ *with the antisense probes indicated above each panel. Dorsal views, anterior to the top. Red arrowheads indicate notochord precursors. **Figure S3. Additional expression patterns in *Oikopleura *embryos at 7-8 and 10-12 hpf**. *Oikopleura *embryos at 7-8 hpf and 10-12 hpf hybridized *in situ *with the antisense probes indicated at the bottom right of each row. Insets in (A,B) show high magnification views of the regions of the tail showing hybridization signal. Black arrows indicate subchordal cells, green arrowheads indicate the developing ciliary funnel.

Considering the morphological similarities between the notochords of *Ciona *and *Oikopleura*, it seems surprising that the *Oikopleura *genome is apparently lacking roughly half of the notochord genes that we searched for, and that only a fraction of the *Ciona *notochord genes that possess identifiable counterparts (*i.e*., that are not divergent to the point of not being recognizable by our searches) in *Oikopleura *are expressed in the notochord. The *Ciona *notochord genes that we used as a reference for this study are active in many cellular processes; genes of still unknown function comprise the largest percentage, while genes encoding transcription factors and other nucleic acid-binding proteins make up the smallest portion (Figure. 8A). Only 21% of the genes of still unknown function (*Ciona Noto *genes; [59]) were found in the *Oikopleura *genome, and only some of them are expressed in the notochord (Figure. 8B). Genes involved in cellular metabolism and replication represented the largest category of *Ciona *notochord genes for which we found orthologs in the *Oikopleura *genome, although only 37.5% of them were expressed in the *Oikopleura *notochord (Figure. 8B).

It has been demonstrated by previous studies that extracellular matrix proteins are crucial for notochordal integrity [46,47] and we have previously shown that extracellular matrix genes are highly conserved between the notochords of vertebrates and *Ciona *[13]. Only seven of the *Ciona *notochord genes included in this study encode extracellular matrix proteins (Figure. 8A); of these, four are present in the *Oikopleura *genome, and are indeed expressed in the *Oikopleura *notochord, either homogeneously, as in the case of *Od-thrombospondin 3*, *Od-FCol1*, and *Od-leprecan *(Figure. 4G-L, AB-AD), or in an unusually restricted pattern, as in the case of *Od-laminin α1 *(Figure. 5K-M). These data might tentatively suggest that either fewer extracellular matrix proteins are required in *Oikopleura *for notochordal integrity, or, perhaps more likely, that in *Oikopleura *different extracellular matrix genes, which are not necessarily found or are still undescribed in the *Ciona *notochord, have been co-opted to this structure. Nevertheless, the similarity in the expression pattern of *Od-FCol1 *in notochord and muscle cells, which is directly comparable to that of the *CiFCol1 *pattern [60,61], seems a strong indication of the requirement of fibrillar collagen for the structural role of the notochord, and possibly of its flanking muscle [62].

### First insights into the *cis*-regulatory mechanisms controlling notochord gene expression in *Oikopleura*

A comparison between the expression patterns of the *Oikopleura *notochord genes identified by this analysis and the published expression pattern for *Oikopleura Brachyury *(*OdiT*) reveals considerable differences, at both the spatial and the temporal level. Differently from *Ci-Bra*, which is notochord-specific at all stages [10], *OdiT *is expressed in a notochord-specific fashion only at early embryonic stages, while in larvae incubated at 12°C and fixed 30 minutes after hatching (roughly corresponding to the late Stage 1 in this study) expression begins to expand to endodermal territories in the trunk and to slowly decline in notochord cells [30]. Finally, in larvae one hour after hatching (approximately Stage 2 in this study), *OdiT *is still strongly expressed in the trunk, but has completely faded in the notochord [30].

Most of the notochord genes identified in this study are expressed in the notochord at mid-tailbud and Stage 1; however, in Stage 2 embryos expression of several of these genes begins to fade in notochord cells, and in older embryos all these genes become no longer detectable. This suggests that the temporal regulation of the transcription of these genes is differentially modulated in *Oikopleura*, possibly through the interplay of more activators that might be themselves transiently expressed in notochord cells. A complementary scenario would imply that late-onset transcriptional repressor(s) begin counteracting the function of the notochord activator(s) by the time Stage 2 is reached. In either case, it is still possible that OdiT might be (co-)regulating the transcription of at least some of the genes described here in notochord cells. This hypothesis seems supported by the observation that the genomic loci of most of the newly discovered *Oikopleura *notochord genes contain various putative Brachyury binding sites matching the core sequences previously identified in *Ciona *[14,34,63], although their distribution is quite variable and does not mirror that of the binding sites found in the Ci-Bra direct target genes (data not shown). Therefore, the notochord genes identified through this analysis are likely to provide a useful platform for future studies of the molecular mechanisms through which notochord gene expression is controlled in *Oikopleura*.

## Conclusions

Tunicate embryos provide excellent model systems for studies of notochord development and evolution. Here we have compared the molecular toolkits of the simple notochords of two divergent tunicates, *Ciona intestinalis *and *Oikopleura dioica*, and we have begun to make inferences about the commonalities and discrepancies among their genetic makeup. Based only upon the low conservation of the 50 notochord genes that we analyzed in these two tunicates, it might be concluded that significantly less genes than expected are required for notochord formation in *Oikopleura*. However, since the *Oikopleura *genome is considerably divergent from those of other tunicates, it seems reasonable to assume that other genes might fill the roles played by the non-conserved genes. In fact, despite the apparent absence from the *Oikopleura *genome of a large fraction of the notochord genes found in *Ciona*, which parallels the striking lack of genes required for the metabolism of retinoic acid [64], we have found that two of the genes that are single-copy in *Ciona*, *Noto15 *and *Noto9*, are duplicated in *Oikopleura*. Gene losses accompanied by duplications of some of the remaining genes seem to match a general genome-wide trend in *Oikopleura *[37], which has been previously observed in the divergent and compact genome of *C. elegans *(e.g., [65]). It has been hypothesized that retention of duplicates can be causally linked to genome compaction [37]. The retention of duplicate genes might also explain why, despite its smaller size and the lack of several genes that are found in *Ciona*, the *Oikopleura *genome is estimated to contain approximately 18,020 genes ([22,37], a number comparable to that assessed for *Ciona *[66].

The results of the expression studies presented here suggest that the gene duplications that we have uncovered may have included to a variable extent the *cis*-regulatory modules (CRMs) controlling the expression of the duplicated genes. For example, *Od-Calumenin1 *and *Od-Calumenin2 *are both expressed in the *Oikopleura *notochord, although they lie on different scaffolds, suggesting, among other scenarios, the possibility that their notochord CRM(s) were duplicated along with their coding regions. However, *Od-Calumenin1 *is expressed in the notochord at all stages tested, while *Od-Calumenin2 *expression is curtailed by Stage 2, which in turn indicates that the temporal regulation of the notochord expression of these genes has changed. Similar differences are observed when the expression patterns of the *Od-Noto9 *paralogs are compared, whereby the expression patterns of *Od-Noto9b *and *Od-Noto9c *are practically overlapping and *Od-Noto9a *seems to have a later developmental onset.

In higher chordates, the notochord is known to serve a dual function: providing structural support to the developing embryo, and secreting patterning signals required for tissue specification and organogenesis [1]. The conserved expression of *netrin *in *Ciona *[54] and in *Oikopleura *suggests that the tunicate notochord, in addition to its structural role, is able to provide some essential positional cues to the developing nervous system. We included in this study also four genes that we had previously found to be expressed in both the *Ciona *and mouse notochord, namely *ASAK*, *leprecan*, *pellino *and *Noto2 *[15]. Interestingly, *Od-leprecan *was also expressed in the *Oikopleura *notochord, while *Od-ASAK *was undetectable in this structure at all the stages analyzed. No signal was obtained for *Od-pellino*, and no ortholog was identified in *Oikopleura *for *Noto2*.

These observations, together with the remarkable absence from the *Oikopleura *genome of *Sonic hedgehog *(D. Chourrout, personal communication) and previous observations that in *Ciona *neither one of the two *hedgehog*-related genes seems to be expressed in notochord cells [67], begin to pinpoint the molecular pathways that are already present in the simple tunicate notochord, as opposed to vertebrate innovations. *Oikopleura *and *Ciona *therefore provide us with a model for investigating the role of mechanisms such as co-option and divergence in notochord evolution and for ascertaining the extent of modifications that its genetic toolkit can sustain without major morphogenetic changes. By comparing the essential complement of genes employed by these divergent tunicates to build their simple notochords, we can begin to envision how notochord structure and function have changed during chordate evolution.

## Methods

### Animal collection and culture

*Oikopleura dioica *adults were collected in fjords near Bergen (Norway) and maintained in culture in plastic beakers at 15°C [23]. *In vitro *fertilizations were set up to collect embryos for WMISH; females were collected in watch glasses, washed with artificial seawater and left to spawn. Sperm from 3-5 males was checked for viability and used for fertilization. The resulting embryos were allowed to develop at 21°C and collected at the time-points specified in the text.

### Identification of *Oikopleura *orthologs of *Ciona *notochord genes

Putative *Oikopleura *orthologs of Ci-Bra target genes were retrieved using BLASTX and TBLASTN algorithms [38] on the current assembly and translated gene models from the *Oikopleura *genome ([37] and http://www.genoscope.cns.fr/externe/GenomeBrowser/Oikopleura/).

Additional sequences from chordates of interest were retrieved through the BLASTX, TBLASTN and BLASTP algorithms using the respective genome and/or ESTs browsers. The reciprocal best BLAST hit method was used to select putative orthologous sequences. Whenever necessary, existing gene models were individually verified by aligning genomic and EST sequences using the BioEdit sequence alignment editor [68].

### Phylogenetic analyses

Multiple alignments of deuterostomes sequences were performed using the MUSCLE 3.6 software and modified manually whenever necessary [69]. Neighbor-joining analyses were done using the BioNJ algorithm [70] and 1000 bootstrap replicates on MEGA version 4.0 [71]. Bayesian inferences were performed on the Phylogeny.fr platform (http://www.phylogeny.fr/) [72] using the method implemented in the MrBayes (v3.1.2) program [73,74]. The number of substitution types was fixed to six. We used the WAG substitution frequency matrix [75], while rates variation across sites was fixed to "invgamma". Four Markov Chain Monte Carlo (MCMC) chains were run for 100,000 generations (sufficient to obtain chain convergence), sampling every 10 generations, with the first 2500 sampled trees discarded as "burn-in". Marginal probabilities at each internal branch were taken as a measure of statistical support.

### Probe Preparation

All probe templates were amplified by RT-PCR as previously described [55] using cDNA prepared from 4-5 hpf *Oikopleura *embryos. A complete list of the primers used can be found in Additional file 3. Plasmids were purified using the QIAprep Spin Miniprep kit (Qiagen, Valencia, CA, USA), linearized using appropriate restriction enzymes (New England Biolabs, Ipswich, MA, USA), then cleaned by standard phenol-chloroform extraction followed by ethanol precipitation. 1 μg of each purified plasmid DNA was used as a template for *in vitro *transcription of antisense RNA probes in the presence of 11-digoxigenin-UTP (Roche, USA), according to the manufacturer's instructions. Probes were purified by lithium chloride precipitation, then resuspended in 50% formamide, 5x SSC, 500 μg/ml yeast tRNA, 50 μg/ml heparin, 9.2 mM citric acid, and 0.1% Triton X-100, and stored at -20°C.

### Whole-mount *in situ *hybridization (WMISH)

Embryos were fixed in 4% paraformaldehyde, 0.1 M MOPS (pH 7.5), and 0.5 M sodium chloride overnight at 4°C, then washed in 0.1 M MOPS (pH 7.5) and 0.5 M sodium chloride, and stored in 70% ethanol at -20°C until use. Hybridization and detection of probes were performed as in [29] with the following modifications: the protease K treatment was extended to three minutes and was followed by incubation in 4% paraformaldehyde; the RNase A digestion was omitted; the antibody incubation was performed at 4°C overnight.

## Abbreviations

bp: base pair(s); cDNA: complementary DNA; EST: Expressed Sequence Tag; hpf: hour(s) post-fertilization; kb: kilobase(s), or 1000 base pairs; Mb: megabase(s); MOPS: 3-(N-morpholino) propanesulfonic acid; PCR: Polymerase Chain Reaction; RT: reverse transcriptase; SSC: saline-sodium citrate solution; tRNA: transfer RNA; WMISH: whole-mount *in situ *hybridization.

## Authors' contributions

JEK performed BLAST searches, cloned and synthesized probes for WMISH, performed WMISH, and drafted portions of the manuscript. PK performed BLAST searches, carried out the phylogenetic analyses, cloned and synthesized probes for WMISH, performed WMISH, and drafted portions of the manuscript. J-MB oversaw the *Oikopleura *culturing, and collected and fixed embryos. DJ supervised experiments and edited the manuscript. ADG designed the study, supervised experiments and wrote the manuscript. All authors read and approved the final manuscript. The order of the co-first authors was decided by the co-first authors and approved by all other authors.

## Supplementary Material

Additional file 1 **Table 1**. Ci-Bra target notochord genes and their *Oikopleura *counterparts found by the reciprocal best BLAST hit method. Click here for file

Additional file 2 **Figure S1**. Protein sequences used for phylogenetic reconstructions. Click here for file

Additional file 3 **Table 2**. Sequences of the oligonucleotides used as primers in the PCR amplifications. Click here for file

Additional file 4 **Table 3**. Summary of the *in situ *hybridization results. Click here for file

Additional file 5 **Figure S2**. Whole-mount *in situ *hybridization of *Oikopleura *neurulae. Click here for file

Additional file 6 **Figure S3**. Additional expression patterns in *Oikopleura *embryos at 7-8 and 10-12 hpf. Click here for file
