# Supplementary material for: Evolutionary changes in the notochord genetic toolkit: a comparative analysis of notochord genes in the ascidian Ciona and the larvacean Oikopleura
Source: BMC Evol Biol. 2011 Jan 20;11:21. doi: 10.1186/1471-2148-11-21 (PMC3034685; doi:10.1186/1471-2148-11-21)
Supplement: Additional file 2 — Figure S1. Protein sequences used for phylogenetic reconstructions. [file 1471-2148-11-21-S2.PDF]

Noto 15 Multiple Alignment

```
                             10        20        30        40        50        60        70        80        90       100
                    ....|....|....|....|....|....|....|....|....|....|....|....|....|....|....|....|....|....|....|....|
Hs-Ran              -------------------------------------------------------MAAQGEP-----------------------------------QVQ
Ci-Ran              --------------------------------------------------------------------MQQQQQD----------------------DLV
Ci-Rholike1         -------------------------------------------------------------------------------------------------MQP
Ci-Rholike2         -------------------------------------------------------------------------------------------------MQS
Bf-XP_002610920.1   ------------------------------------------MASASKNPFLEEGVRMSVDPIKLKEVENDVRTG-------------LCKFTE---REP
Bf-XP_002601232.1   ----------------------------------------------------------MAVGNAVDNLRENVRDR-------------IRNAS----AEM
Bf-XP_002614007.1   -------------------------------------------------------MAQGPKPGELESLRDKVKKR-------------LQNIP----GKE
Bf-XP_002614002.1   --------------------------------------------------------MAEAQGMSLNELRDRVKSR-------------LTHTLHDQPGKK
Bf-XP_002599629.1   --------------------------------------------------------MAQYEPIPVSSLRDQVKQR-------------LTGMP----KKE
Ci-Noto15           ---------MAPQTASTGNVNLINGRNAVSASKELLDCNHNAVISEKAAQQQATVGAPNKRPSNGGDVNDRSRKL-------------IKSCFTHL-NTN
Cs-Noto15           ---------MAPQTASTGNVNLINQQSTLAASKEHLDCNHNAVISE-KTPQQATVTASSKRPSVGGDVNDRSRKL-------------IKSCFTHL-NTN
Hr-Noto15           -------------------EVLSVEPSTEAPEC-------------CEKPAAERNEVFKPASVPNRTINDRTRKM-------------IENMFNKHLSTN
Mt-Noto15           ----------------------------------------------------------------------------------------------------
Dl-Noto15           TLTVMCKVTSMIVERSASVEDLAMAPLREEPMQGEESCLAVNPGCDCNNNNSSGMETGSKRPSNSGDVNERSRKL-------------IKSCFVTL-TSN
Od-Noto15a          ---------------MKRNASHKMIYDADEESGFDEIPEEVFEGETSQAPGFLAGLQHRESIQAEKNNLDRYNHT-------------IKSALCDL-CEP
Od-Noto15b          -------MSEIDILEQPVFEEETFDSSSSSPSTSPKPLMIPHQRHPRQRYNSSSIRRTPVVPGYVSELIERRESLRVLPEGRSKDNTCIRKALQYI-ADP

                            110       120       130       140       150       160       170       180       190       200
                    ....|....|....|....|....|....|....|....|....|....|....|....|....|....|....|....|....|....|....|
Hs-Ran              FKLVLVGDGGTGKTTFVKRHLTGEFEK-----KYV---------------ATLGVEVHPLVFHTN-----------------------------------
Ci-Ran              VKLVLVGDGGVGKTTFVKRHLTGEFEK-----KYV---------------ATLGVEVHPIVF------------------------------------QT
Ci-Rholike1         VKIVVVGDGAVGKTCMCITYANGVFPS-----DYV------------------PTIFDNYAA--------------------------------TSTFEG
Ci-Rholike2         IKLVVVGDGAVGKTCLLISYTANAFPR-----EYV------------------PTVFENYMA----------------------------------NITV
Bf-XP_002610920.1   MNVLVIGKPGAGKSSFINSMHMAIAHRWYEVANYG-D-----------GRRGITTDLIRYDMFRDELT--------------------------EPPIKG
Bf-XP_002601232.1   FNIVFIGLTGSGKSSLINSMEQAVVGTPANLARVGRG-----------AGGNVTLCLEGCEMFST-------------------------------LQDN
Bf-XP_002614007.1   FNVLVVGMYGSGKSSFINSMVMAVTGTWHEYAHYANA-----------PYLSVTKRLERYVMFDDKCKEEDE----------------------KHSIPG
Bf-XP_002614002.1   LNVLVVGMPGSGKSSYINSMIMAVTGTWHEIARYGRTASTHEMAQRLVGESRVTSCLESFVMFDDNCHEVGE----------------------SHPMPD
Bf-XP_002599629.1   FNVLVVGVPGSGKSSFVNSMIMAVTGTWHEVAHYSER-----------FSGRATHSLERYVMFDDNCKEVHE----------------------PHPIPD
Ci-Noto15           LNVLVIGAKGAGKSSLINSMNMALTQEWKDRAKYC---------------PGRNHVIDECVMFHN---------------------------------KK
Cs-Noto15           LNVLVIGAKGAGKSSLINSMNMALTQEWKDRAKYC---------------PGRNHVIDECVMFHN---------------------------------KK
Hr-Noto15           LNVLVLGQRESGKSSFINSMAMSLSQKWSDKAKYC---------------PGRNHIIDECVMHKN----------------------------------T
Mt-Noto15           ----------------------------------------------------------------------------------------------------
Dl-Noto15           LNVLVVGSKNSGKSSLINSMNMALTQQWIDRARYC---------------PGRTHVIDQCVLYHN-----------------------------RKEKKT
Od-Noto15a          LNVIVIGPHDSGKTTVINSLLMSVRGKWTDRAKYG---------------HGSAHQLSPVLVYENPNHHHQN-----------CPEHKHYDPHWKRPQHV
Od-Noto15b          LNVLVIGPHDSGKTTLINSLLMSVTGEWVDRAPYG---------------TGRAHNLNPVVLYENPEHHRRHRTVSDANNGGYRPRHRHFDPTWKRSCED

                            210       220       230       240       250       260       270       280       290       300
                    ....|....|....|....|....|....|....|....|....|....|....|....|....|....|....|....|....|....|....|
Hs-Ran              -RGPIKFNVWDTAG-----QEKFGGLRDGYYIQAQ-----CA--IIMFDVTSR---VTYKNVPNWHRDLVRVCENIPIVLCGNKVDIKDRKVKAKSIVFH
Ci-Ran              QRGRIRFNVWDTAG-----QEKFGGLRDGYYIQGQ-----CA--IIMFDVTSR---VTYKNVPNWHKDLVRVCEDIPIVLVGNKVDIKDRKVKAKAINFH
Ci-Rholike1         ISYNLAL--WDTAG-----QEEYDRLRPLSYPRTNVFLL-CF--SVVSPASFF---S---ISSKWHPEVSHHSPKTPCILIGTKQDLRDDEETVKKLKNT
Ci-Rholike2         NNQQICLSLWDTAG-----QEDFDRLRPLSYPDTDVFVL-CF--SIISPTSFE---N---LQHKWLPELREHCPNVPILLVGTKLDLREDTEILQQLSSK
Bf-XP_002610920.1   YNHGVFF--WDTAGLEDLNQKIFETFL-GLIMEGRIPPDTNI--HEHTDTTRQRPPTVSSLKRRFPRSGAVEEWKCHRIVFLCAADEEPPMNLMEAVTDA
Bf-XP_002601232.1   LRDKVKF--WDFVGLPNRNDETIRTLI-SLALDGRIPENQPLINPEYFEMCKE---E---LEDEFPAV--QGGPTIQRVVVVCAADEEMPVNLLQATKKA
Bf-XP_002614007.1   YRKNVIF--WDCPGFPDASEEAYSTIV-SLTLDGRIPPGTKV--LEFINQSPE---Q---LRNKFGRV--NRKMTFDRIVYINSADCDVHKLLVTAIKSG
Bf-XP_002614002.1   YNKNIVF--WDCAGFPDATEEVYSTIV-SLTLDGRIPSGTNV--IECMDQTPD---Q---LRSKFRRV--DRQMTFDRIVFVLSADKMVPYNLVEAIRSG
Bf-XP_002599629.1   YKQNVIF--WDCAGFPNANDEAYSTIV-SLTLDGCMPTD------------------------------------VPIVLVVTKIDKCPEQDLQQRIEEA
Ci-Noto15           AGGKVVF--WDTRGFEDIHADDHAILILRYVLEGRIPAK-CI--PCVLLMSKE---I---IKKRYHKI-SETKRRIDLVLFVSSLTEEPNTRLMSLTEQA
Cs-Noto15           AGGKVVF--WDTRGFEDIHADDHAILILRYVLEGRIPAK-CI--PCVLLMSKE---V---IKKRYHKI-SETKRRIDLVMFVSSLTEEPNSRLMSLTEQA
Hr-Noto15           SGGKVVF--WDTRGFEDIHELDHAVLILRYVLEGRIPAK-CI--PCVLLMNKE---L---IKKRYHRT-FDSNRRIDCILFVSALDEDPPTHLMSIVQQA
Mt-Noto15           -----------------VHNEDKAVLILRYVLEGRIPAK-CI--PCVLLMEKE---L---IKKRYHKI-YESDRRIDCVLFVSALDEEPPKRLMTLLQKA
Dl-Noto15           SDGKVVF--WDTRGFEDIHSIDHATLILRYVLEGRIPPK-CI--PCVLLMSKD---L---IKKRYQKI-TSSDRRIDLVLCVSALDQEPQTRLMGIVQNA
Od-Noto15a          SCGRVTF--WDTRGFEKIADKEKAALILRYILEGRLNFR-LF--TQALLMEKE---E---IKR--IRE-SNRDLQIDLILYVASAKEKPNLELFDVIQNA
Od-Noto15b          PCGRVVM--WDTRGFDTIHDEAHQALLLRYILEGRLHPS-NL--SQALLLPDE---I---CKKRYRAP--MKDSQIDMILYVAAADERPNVNLFKAIIRA

                            310       320       330       340       350       360       370       380       390       400
                    ....|....|....|....|....|....|....|....|....|....|....|....|....|....|....|....|....|....|....|
Hs-Ran              RKKNLQYYDISAKSNYNFEKPFLWLARKLIGDPNLEFVAMPAL-----------------------------APPEVVMDPALAAQYEHDLEVAQTT---
Ci-Ran              RKKNLQYYDISAKSNYNFEKPFLWMARKLMGDPNLEFCAMPAM-----------------------------LPPEVKLDDNLMKQYEEELQNAQQC---
Ci-Rholike1         DSAPI---------------------RYEQGEALAKQLGAVKYVE-----------------------------CSALKKVGVDSVFQDAISAVLNP---
Ci-Rholike2         NLKPI---------------------TPEEGAKMAK------------------------------------DIKAVKYLECSALTQECLSQVFDDA---
Bf-XP_002610920.1   ATKDKADARILDLRIFQHLMLRVKVLKLAANETHRNTCCLMSHTTQEPSSQIAELEDEGGNNTCMGQGISCVPVPKRRRRPTVCITACSTTTLYAVENLV
Bf-XP_002601232.1   AQPAT-TASDRNIPLFLLISKVDKMVGDANKDDLSRRIRSARD-----------------------------TLDATNYFFEKATLYHEQTNRDNDPGSR
Bf-XP_002614007.1   AQKDH------DIPIFAMMTKID------KADDLQKRVEDAQAAL---------------------------TLTDNNVRFKTTSLYCKDVTACTDP---
Bf-XP_002614002.1   AEKGH------DVPFIVLVTKVD---KCPNPHDLQQRVLGAQAAF---------------------------NLIGHQGRFKISSLYCEDVTPWPDP---
Bf-XP_002599629.1   KGAT--------------------------------------------------------------------DLKGNRVRFKKTSLYCEELTSFPDP---
Ci-Noto15           ITTSK-HTCVNRVPIVSVATKSDLFNTVKPDELHDYSLEHVVTERRDSARASRRSRLA--------------TVPEVVASAKVVDNYRCELEPWSDE---
Cs-Noto15           ITTSK-HACVNRVPILSIATKGDMFTSLKPDVLHDYSLEHVLNERRVFSHTSRRSRLA--------------PVPEVVAGAKIVTNYRCELEPWSDE---
Hr-Noto15           VATSK-HVSVNRIPIISATTKSDLL-STERVEQLENSRGYLNYSLEHYVSRN--------------------DPNRMEYNPRRVVNYSCELEPFSDE---
Mt-Noto15           VQTSK-HVSVNRVPMISVITKKDSLKQNEADEKLNRIAEYNLS---------------------------------PDFSTRIVENYRCELDSWSDE---
Dl-Noto15           IDSSK-----------------------------------------------------------------------------------------------
Od-Noto15a          RNDSK-LPSVRSVPILTALTKYDLLSSEEIIRVDEEIKNYHPNSLNQG------------------------NLPEEALTPYHVIAYNSKVNPIDDE---
Od-Noto15b          KEESK-DEKAMKCPIMLVMTKFDLLDEVEKAAVNDNIFFYHPSSYGFHGDVFSPDF----------------DLQDEEVLWKKVHCYESNLNPVIDR---
```

```
                            410       420       430       440       450       460       470       480       490       500
                     ....|....|....|....|....|....|....|....|....|....|....|....|....|....|....|....|....|....|....|....|
Hs-Ran               -------------------------ALPDEDDDL------------------------------------------------------------------
Ci-Ran               -------------------------SLPDEDDDL------------------------------------------------------------------
Ci-Rholike1          ------------------------AKPPKPTKRC---------VLL------------------------------------------------------
Ci-Rholike2          ----------------VIAVLNPSHFSSNNDNSC------------------------------------------------------------------
Bf-XP_002610920.1    RAALGESINIDIHTVHIQKRESISLLKKYGSKTYFICCANYGHRLLLLSDTDP-----------------------------------------------
Bf-XP_002601232.1    SRHTMATQDSFHLKCREAKHSDYNAVMKMASVDTFRDGFDYLPAKFHQWVNDPQFHVLVAEADDKVVAVYVCCMADGGTYLFNKTMRVAPDLQGKKLMMR
Bf-XP_002614007.1    --------------------NDYRVMMPNPKIDS---------VLLNTWMN-------------------------------------------------
Bf-XP_002614002.1    --------------------DDYRVMKPNAEIDR---------KLLNIWMN-------------------------------------------------
Bf-XP_002599629.1    --------------------NDYRVMMPNEEIDR---------NLLNIWMS-------------------------------------------------
Ci-Noto15            -------------------SANPSMVKSSPDRDA---------LLLSVWRE-------------------------------------------------
Cs-Noto15            -------------------SVNPSEVKSSPDRDA---------NLLGVWRE-------------------------------------------------
Hr-Noto15            -------------------SANPSSMTPSAERDT---------PLLAVWRD-------------------------------------------------
Mt-Noto15            -------------------SANPSLVHSSSERDS---------PLLAIWRD-------------------------------------------------
Dl-Noto15            ----------------------------------------------------------------------------------------------------
Od-Noto15a           ------------------------FIPPTPDKRR-------NGAMLKLFME-------------------------------------------------
Od-Noto15b           ----------------------LEQNQPDEKKDE---------KLLKLFKD-------------------------------------------------

                            510       520       530       540       550       560       570       580       590       600
                     ....|....|....|....|....|....|....|....|....|....|....|....|....|....|....|....|....|....|....|....|
Hs-Ran               ----------------------------------------------------------------------------------------------------
Ci-Ran               ----------------------------------------------------------------------------------------------------
Ci-Rholike1          ----------------------------------------------------------------------------------------------------
Ci-Rholike2          ----------------------------------------------------------------------------------------------------
Bf-XP_002610920.1    ----------------------------------------------------------------------------------------------------
Bf-XP_002601232.1    IGFDLEKALQLRLHPRLLHLERRGIAKDDMYFQGTCNEVLSSLENKDVLPYSLQSSVVPYQPSDMARLTRPEFVEQILSDNSIVVDTIMYQLSASNLHGL
Bf-XP_002614007.1    ----------------------------------------------------------------------------------------------------
Bf-XP_002614002.1    ----------------------------------------------------------------------------------------------------
Bf-XP_002599629.1    ----------------------------------------------------------------------------------------------------
Ci-Noto15            ----------------------------------------------------------------------------------------------------
Cs-Noto15            ----------------------------------------------------------------------------------------------------
Hr-Noto15            ----------------------------------------------------------------------------------------------------
Mt-Noto15            ----------------------------------------------------------------------------------------------------
Dl-Noto15            ----------------------------------------------------------------------------------------------------
Od-Noto15a           ----------------------------------------------------------------------------------------------------
Od-Noto15b           ----------------------------------------------------------------------------------------------------

                            610       620       630       640       650       660       670       680       690       700
                     ....|....|....|....|....|....|....|....|....|....|....|....|....|....|....|....|....|....|....|....|
Hs-Ran               ----------------------------------------------------------------------------------------------------
Ci-Ran               ----------------------------------------------------------------------------------------------------
Ci-Rholike1          ----------------------------------------------------------------------------------------------------
Ci-Rholike2          ----------------------------------------------------------------------------------------------------
Bf-XP_002610920.1    ----------------------------------------------------------------------------------------------------
Bf-XP_002601232.1    SKRGVTILVDNIKRDLFCAAAADFLINAVSCVTRKSSPRFAHASKLRQQRGMIAVYVCYMTDGGAYLFHKSIRIAPKLQGKKLLTRIGFELEKALQLRLH
Bf-XP_002614007.1    ----------------------------------------------------------------------------------------------------
Bf-XP_002614002.1    ----------------------------------------------------------------------------------------------------
Bf-XP_002599629.1    ----------------------------------------------------------------------------------------------------
Ci-Noto15            ----------------------------------------------------------------------------------------------------
Cs-Noto15            ----------------------------------------------------------------------------------------------------
Hr-Noto15            ----------------------------------------------------------------------------------------------------
Mt-Noto15            ----------------------------------------------------------------------------------------------------
Dl-Noto15            ----------------------------------------------------------------------------------------------------
Od-Noto15a           ----------------------------------------------------------------------------------------------------
Od-Noto15b           ----------------------------------------------------------------------------------------------------

                            710       720       730       740       750       760       770       780       790       800
                     ....|....|....|....|....|....|....|....|....|....|....|....|....|....|....|....|....|....|....|....|
Hs-Ran               ----------------------------------------------------------------------------------------------------
Ci-Ran               ----------------------------------------------------------------------------------------------------
Ci-Rholike1          ----------------------------------------------------------------------------------------------------
Ci-Rholike2          ----------------------------------------------------------------------------------------------------
Bf-XP_002610920.1    --------------------------------------------------DELTEVVYHAGKISKYKKDGVMVLLFGHRNLTEGVYDLSTFDGAFMRSQP
Bf-XP_002601232.1    PRLLHLERHAIADVKRLYLLKGMNRKDICVIASIHLFTVSHDVMYFQGTCNEVLSNLEAKGVLPYPPPPSVMPFQPSDMVRLIRPEFLEQILSDKSIFVD
Bf-XP_002614007.1    ----------------------------------------------------LTDPNIRVIETPPPH---------------------------------
Bf-XP_002614002.1    ----------------------------------------------------LTDPNIKAKPAPPAP---------------------------------
Bf-XP_002599629.1    ----------------------------------------------------LTDRNIKAKPAENES---------------------------------
Ci-Noto15            ----------------------------------------------------IVSRTAACAGNSDRR---------------------------------
Cs-Noto15            ----------------------------------------------------IVSRTAACSGNADRR---------------------------------
Hr-Noto15            ----------------------------------------------------IISRTT------------------------------------------
Mt-Noto15            ----------------------------------------------------IIQRTTACGGADKRK---------------------------------
Dl-Noto15            ----------------------------------------------------------------------------------------------------
Od-Noto15a           ----------------------------------------------------IIALARPNGRRRINS---------------------------------
Od-Noto15b           ----------------------------------------------------IIDYSLTPTGKRRCS---------------------------------
```

```
                        810       820       830       840       850       860       870       880       890       900
                  ....|....|....|....|....|....|....|....|....|....|....|....|....|....|....|....|....|....|....|....|
Hs-Ran            ----------------------------------------------------------------------------------------------------
Ci-Ran            ----------------------------------------------------------------------------------------------------
Ci-Rholike1       ----------------------------------------------------------------------------------------------------
Ci-Rholike2       -----------------------------CKIV-------------------------------------------------------------------
Bf-XP_002610920.1 RLGEKAGRGKFISVYDAFNSDQEECLRRWVRIPTEKDEALGEEPMLMGPTENVEASEEDPFLDKVHENPPLLEPENINEARKEYPRTLQILEIDPHGYND
Bf-XP_002601232.1 WDQYQLSASSLHGLSKRGVTILVDNSEPAAKSLSYGGSYMTPRGRVYHVDVHCSDVGLCQVHIKHHVDRACREYDGVITFCVMVIDPSLIITTKTFCVET
Bf-XP_002614007.1 --------------------EEPPRSNIWCVLL-------------------------------------------------------------------
Bf-XP_002614002.1 --------------------------ESWCTTS-------------------------------------------------------------------
Bf-XP_002599629.1 -----------------------------CCVL-------------------------------------------------------------------
Ci-Noto15         ------------------KRAYSASSRPMCRCLPFKFDYMRLRSASF-----------------------------------------------------
Cs-Noto15         ------------------KRAYSASSRPMCRCLPFRFDYMRLRSASF-----------------------------------------------------
Hr-Noto15         ----------------------------------------------------------------------------------------------------
Mt-Noto15         -----------------RAYSSPNSNRGFCKCLPFRFDYKRLRSGSF-----------------------------------------------------
Dl-Noto15         -----------------------------LRCV-------------------------------------------------------------------
Od-Noto15a        ------------------------ASAIMLRSLSRGFVRRRHRSNSNPRDRFVNA---------------------------------------------
Od-Noto15b        --------------------SSSSKIFHLARNTGQKLLSRRLRTISI-----------------------------------------------------

                         910       920       930       940       950       960
                  ....|....|....|....|....|....|....|....|....|....|....|....|...
Hs-Ran            ---------------------------------------------------------------
Ci-Ran            ---------------------------------------------------------------
Ci-Rholike1       ---------------------------------------------------------------
Ci-Rholike2       ---------------------------------------------------------------
Bf-XP_002610920.1 EYDGPVSTV------------------------------------------------------
Bf-XP_002601232.1 LGLVHLPNDHRYLSYKFMFLRIHVRIGCAKTNRQLIRRLGIEGRIDRLLQEALQQNNRLSPSA
Bf-XP_002614007.1 ---------------------------------------------------------------
Bf-XP_002614002.1 ---------------------------------------------------------------
Bf-XP_002599629.1 ---------------------------------------------------------------
Ci-Noto15         ---------------------------------------------------------------
Cs-Noto15         ---------------------------------------------------------------
Hr-Noto15         ---------------------------------------------------------------
Mt-Noto15         ---------------------------------------------------------------
Dl-Noto15         ---------------------------------------------------------------
Od-Noto15a        ---------------------------------------------------------------
Od-Noto15b        ---------------------------------------------------------------
```

Noto 9 Multiple Alignment

```
                     10        20        30        40        50        60        70        80        90       100
            ....|....|....|....|....|....|....|....|....|....|....|....|....|....|....|....|....|....|....|....|
Ci-HNRNPK   --------------------------------------------------------------------------MSEATDSSTN----------------
Sp-HNRNPK   --------------------------------------------------------------------MSEDQDYGSGEGG-------------------
Hs-HNRNPK   ----------------------------------------------------------------------METEQPEETFPNTETNGEFG----------
Mm-HNRNPK   ----------------------------------------------------------------------METEQPEETFPNTETNGEFG----------
Sk-HNRNPK   --------------------------------------------------------------------MQDPALMGDDGALGMQDQQEGN----------
Bf-HNRNPK   --------------------------------------------------------------------MESEPINSDEFKQERDSWDQEG----------
Od-Noto9c   --------------------------------------------------------------------MDHNMPILNDSGVTGQ----------------
Od-Noto9a   ---------------------------------------------------------------------MGVNLFNEVTTR-------------------
Od-Noto9b   ---------------------------------------------------------------------MNGIENGNNLKR-------------------
Ci-Noto9    --------------------------------------------------------------------------MGDAPN--------------------
Cs-Noto9    --------------------------------------------------------------------------MGDAPN--------------------
Mt-Noto9    --------------------------------------------------------------------------MGDFSQ--------------------
Hr-Noto9    --------------------------------------------------------------------------MGDYSQ--------------------
Sp-FUSEBP   MTDYTSATPQQSTT------------------------------------------------------LDGNAAFADAVQRARAIAAKIGGSTTLGLNS-
Pl-FUSEBP   MTDYTSATPQQSTT------------------------------------------------------LDGNAAFADAVQRARAIAAKIGGSSTLGLNS-
Bf-FUSEBP   MMDYSAVAPPSAMSTP----------------------------------------------------ETQNAAFADALQRARQIAAKIGGDGAVPETDS
Sk-FUSEBP   MSDYNAVAPPAAP-------------------------------------------------------IDTNAAFADALQRARQIAAKIGGSGTGAPPGT
Mm-FUSEBP3  MAELVQGQSAPV--------------------------------------------------------GMKAEGFVDALHRVRQIAAKIDSIPHLNN---
Hs-FUSEBP3  MAELVQGQSAPV--------------------------------------------------------GMKAEGFVDALHRVRQIAAKIDSIPHLNN---
Pm-FUSEBP   MASQG---------------------------------------------------------------GIKPDAFAEAIQRARQIAAKLGVD-----PGT
Mm-FUSEBP1  MADYSTVPPPSSGSAGGGGG------------------------------------------------GVVNDAFKDALQRARQIAAKIGGD--------
Hs-FUSEBP1  MADYSTVPPPSSGSAGGGGGGGGG--------------------------------------------GGVNDAFKDALQRARQIAAKIGGD--------
Mm-FUSEBP2  MSDYNTGGPPPGPPPPAGGGGGAAGAGGGPPPGPPGAGDRGGGGPGGGGPGGGGASGGPSQPPGGGGPGIRKDAFADAVQRARQIAAKIGGD--------
Hs-FUSEBP2  MSDYSTGGPPPGPPPPAGGGGGAGGAGGGPPPGPPGAGDRGGGGPGGGGPGGGSA-GGPSQPPGGGGPGIRKDAFADAVQRARQIAAKIGGD--------

                    110       120       130       140       150       160       170       180       190       200
            ....|....|....|....|....|....|....|....|....|....|....|....|....|....|....|....|....|....|....|....|
Ci-HNRNPK   ---------------------------------KRSHDDVR--------------ESGDSDVKRSRSS--FQHDRFHQGGREPS----------------
Sp-HNRNPK   ---------------------------------KRSAED-------------------QRQNQNKRPR--GDY---------------------------
Hs-HNRNPK   ---------------------------------KRPAED----------------MEEEQAFKRSRNT--D-----------------------------
Mm-HNRNPK   ---------------------------------KRPAED----------------MEEEQAFKRSRNT--D-----------------------------
Sk-HNRNPK   ---------------------------------KRPADEEI--------------GN-SQSSKRTKGG--GDG---------------------------
Bf-HNRNPK   ---------------------------------KRPAED-----------------EGGQSNKRPRPP--------------------------------
Od-Noto9c   ---------------------------------KRPSDNAA--------------ENNGPTAKKANTA--NLEVVKDEG---------------------
Od-Noto9a   ---------------------------------ETFRTGAQ--------------GVVTPSVSSMNGL--NPPQSHPIP---------------------
Od-Noto9b   ---------------------------------ERGMEDFG--------------ASGAPQLYTQDGP--PDAKRPMNDVFGGE----------------
Ci-Noto9    ---------------------------------KRPLED----------------GE-EPGPKKLATP--QTSAANSEQIEFDNLGMQQNP---------
Cs-Noto9    ---------------------------------KRPLED----------------GE-EPGPKKLATP--QTFAANSEFAESNKLGMQENP---------
Mt-Noto9    ---------------------------------KRPLED----------------AE-EPGAKKLASSQLDANMVNSMSGMGG-----------------
Hr-Noto9    ---------------------------------KRPLED----------------AE-EPGAKKLASQ--NDSIGSQLGNMQNQIPGGR-----------
Sp-FUSEBP   -----GSSSAELGGLPPVGGLGALSGGDAASGIKRPLEDSN--------------SDAEPATKNLAMQ--NDNDFNTFGAALSGMPRAAPANSMGGL---
Pl-FUSEBP   -----GSSSAEIGGLPPAGGLGALSGGDAASGIKRPLEDSN--------------SEAEPATKNLAMQ--NDNDFNTFGAALTGMPRAAPANSMGGP---
Bf-FUSEBP   R--------------------------------KRGLEDGPGELNGTPDNMFRDYGDGEPEPKKLAAQ--NDPIGAQLAQQLAQQRNFSSSRAG------
Sk-FUSEBP   VAPPVPASAMPPIGIPGNVDLGGM-------GIKRPLED----------------AEGEPDSKKLAAK--NDPIGAQLAALAQQARRHLGNGNQDEFERP
Mm-FUSEBP3  ---------------------------------STPLVD----------------PSVYGYGVQKRAL--DDGVGNQLGALVHQR---------------
Hs-FUSEBP3  ---------------------------------STPLVD----------------PSVYGYGVQKRPL--DDGVGNQLGALVHQR---------------
Pm-FUSEBP   GPAIITSPVNPNPPGVSSLGLGAL---------KRPLED----------------GD-QPESKKLASQ--NDIVSAQLAAIAFQHR--------------
Mm-FUSEBP1  ---------AGTSLNSNDYGYGGQ---------KRPLED----------------GD-QPDAKKVPPQ--NDSFGAQLPPMHQQQR--------------
Hs-FUSEBP1  ---------AGTSLNSNDYGYGGQ---------KRPLED----------------GD-QPDAKKVAPQ--NDSFGTQLPPMHQQQSR-------------
Mm-FUSEBP2  -------AATTVNNNTPDFGFGGQ---------KRQLED----------------GD-QPDSKKLASQ--GDSIGSQLGPIHPPPR--------------
Hs-FUSEBP2  -------AATTVNNSTPDFGFGGQ---------KRQLED----------------GD-QPESKKLASQ--GDSISSQLGPIHPPPR--------------

                    210       220       230       240       250       260       270       280       290       300
            ....|....|....|....|....|....|....|....|....|....|....|....|....|....|....|....|....|....|....|....|
Ci-HNRNPK   --DKIELRVLIPSHTAGAIIGKGGANIRDLRQEFFCPFCSTVLRPIQS-SIPLPRIV--SAVAKTIEDAANICGKVVE-----------------ALNER
Sp-HNRNPK   --NQIIFRLMIRSINAGGVIGKGGENIKRLRVDY---DAKVSI-PDCN-G-P-ERIIKI--GTRNVDNAIDCIKDIIPS----------------VGEKK
Hs-HNRNPK   --EMVELRILLQSKNAGAVIGKGGKNIKALRTDY---NASVSV-PDSS-G-P-ERILSISADIETIGEILKKIIPTLEEGLQL------------PSPTA
Mm-HNRNPK   --EMVELRILLQSKNAGAVIGKGGKNIKALRTDY---NASVS-VPDSS-G-P-ERILSISADIETIGEILKKIIPTLEEGLQL------------PSPTA
Sk-HNRNPK   --PKVDLRILLQSKNAGAIIGKGGNNIHRLRTQY---NATVTV-PDCS-G-P-ERILTVTTNQETALSCLLDVIPVLEEYQ-------------------
Bf-HNRNPK   --PPVDLRILLQSKNAGAIIGKQGLNIKRLRSEY---KATVTV-PDCT-G-P-ERILTISADLNTACACLLDIIPVLEDYQ---------------KHYQ
Od-Noto9c   --DKVVEEYPIPDQLVGLVIGRGGENIQRIQAET---NCGIQVQAQST-GSE-NRPCTLTGTPEQLQAAKNALQEVISNGRIKDEQNARDGKTYSGRRPN
Od-Noto9a   --QQIQDNISVPDQLVGLIIGKNGEQINRIQQET---GCRVQIVPNST-GGS-ERPCTLTGTFHQVHHAKQKLNEIITRGG--------------PRENG
Od-Noto9b   --NLTTEEFPVPDAMVGLIIGRGGDQITKIQADS---GCRVAVVPQST-GGT-SRPCTLTGNPEQIEAAKKMLADIITRGSVK------------EGDVF
Ci-Noto9    --DIMSDTIRLPDDLVGLIIGRGGENIMRMQRET---GCRIQITQSIP-GTK-ERPCTLSGTQEQIEVCRNMLNEIISRSQ--------------AGTLG
Cs-Noto9    --DIMSETIRLPDDLVGLIIGRGGENIMRMQRET---GCRIQITQSIP-GTK-ERPCTLSGTPEQIEVCRNMLNEIISRSQ--------------AGTLG
Mt-Noto9    --SVRTEPFSLPDKYVGLVIGKGGETITRLQSDS---GCRVQIAPESPAGSE-ERPCTLTGTPEQINVCSNMLNDILNNAG-----------------QN
Hr-Noto9    --PTITESFRLPDKLVGLVIGKGGENISRMQEQS---GCRIQISQDCQ-GGE-DRPCTLTGTPEEIGQCREMLQETISKA---------------AGNMG
Sp-FUSEBP   --GSINDELLVPDKVVGLIIGRQGQQISSLQSES---GCNIQIAPENG-ISG-DRQVTLTGTPEAVMHAKSLILDIVNKAS-------------------
Pl-FUSEBP   --GSMNDEVLVPEKLVGLIIGRGGAQISALQTES---GCNIQIAPDSG-GAA-ERQVTLTGTPEAVMHAKQLIHEIVAKAN-------------------
Bf-FUSEBP   --GVVTEEYRVPDKMVGLIIGRGGEQITRLQAES---GCKVQMAQDSG-GLP-ERVCTLTGTPPSIEHAKRLIDQIIEKGRSSGA----------TEQPG
Sk-FUSEBP   GQSSLSEEYAVPDKLVGLVIGRGGEQITRLQAES---GCKIQIAQDSG-GLP-DRMCTLTGNPQAIERAKALIDRIIERGQ--------------GPAVG
Mm-FUSEBP3  --AVITEEFKVPDKMVGFIIGRGGEQISRIQAES---GCKIQIASESS-GIP-ERPCVLTGTPESIEQAKRLLGQIVDRCR---------------NGPG
Hs-FUSEBP3  --TVITEEFKVPDKMVGFIIGRGGEQISRIQAES---GCKIQIASESS-GIP-ERPCVLTGTPESIEQAKRLLGQIVDRCR---------------NGPG
Pm-FUSEBP   --PQMSIEYKVPDKMVGFIIGRGGENISRIQSDS---GCKVQFAPDSG-GQA-DRPCTLTGTPEAIAEAKRMLDQIVERGRIP------------PGPAG
Mm-FUSEBP1  --SVMTEEYKVPDGMVGFIIGRGGEQISRIQQES---GCKIQIAPDSG-GLP-ERSCMLTGTPESVQSAKRLLDQIVEKGR---------------PAPG
Hs-FUSEBP1  --SVMTEEYKVPDGMVGFIIGRGGEQISRIQQES---GCKIQIAPDSG-GLP-ERSCMLTGTPESVQSAKRLLDQIVEKGR---------------PAPG
Mm-FUSEBP2  --TSMTEEYRVPDGMVGLIIGRGGEQINKIQQDS---GCKVQISPDSG-GLP-ERSVSLTGAPESVQKAKMMLDDIVSRGR--------------GGPPG
Hs-FUSEBP2  --TSMTEEYRVPDGMVGLIIGRGGEQINKIQQDS---GCKVQISPDSG-GLP-ERSVSLTGAPESVQKAKMMLDDIVSRGR--------------GGPPG
```

```
                   310       320       330       340       350       360       370       380       390       400
            ....|....|....|....|....|....|....|....|....|....|....|....|....|....|....|....|....|....|....|....|
Ci-HNRNPK   MHHPE-----------------KVGC-------LRMLVHKSQAGTIIGLKGSRIKELREMTGANIKVNQE-CCPESTDRVCQVRGTADVVVKCVARILEH
Sp-HNRNPK   HSQDQ-----------------QNNS------FIRIMVHQSHAGAIIGRAGFKIKELREKTGAHFKVYTE-TCPKSTDRVVQLTGSPDVIAKAAREVYEI
Hs-HNRNPK   TSQLPLESDAVECLNYQHYKGSDFDC------ELRLLIHQSLAGGIIGVKGAKIKELRENTQTTIKLFQE-CCPHSTDRVVLIGGKPDRVVECIKIILDL
Mm-HNRNPK   TSQLPLESDAVECLNYQHYKGSDFDC------ELRLLIHQSLAGGIIGVKGAKIKELRENTQTTIKLFQE-CCPHSTDRVVLIGGKPDRVVECIKIILDL
Sk-HNRNPK   QYKDL-----------------DFDC------EMRMLVHQSQAGAIIGRAGFKIKELREQTGANIKVYSE-VCPNSTERVVQMNGSPEVVINCMRMILDV
Bf-HNRNPK   EHKDL-----------------NFNC------ELRMLVHQSQACCIIGRAGFKIKELREQTEANIKVYSE-CMPGSTERVVALTGKPEKCVGAIKKIIEL
Od-Noto9c   REQNDSRNRAPNTRRAVTQPPMPNPSQPWQTTCEEFLISPDKVGVVIGKGGQNLKNLRQKYQVSLELVQKDTDPEGVAKVLKITGNSTQINGTRMDIFTT
Od-Noto9a   MSYGENKH--------------QGQM------QHEMQIPPDKCGLIIGKGGNTLKQLMQEFNVKLHLVQESAEITRDEKPLKIIGTPQAVESAKNAIVGL
Od-Noto9b   NGHPAPSANDLMNQNVAATASLDGNC------MEEIIIPHDKCGIVIGKAGNTLRNLRSQFGCSVNL--DSTVNTGDPKPLRIAGPPDKVNLVVAEVHKM
Ci-Noto9    SNFNLSQGGLGGMG--------DGGM----EKSIEIAVPPDKCGLIIGKGGETIKMLQQSLGVKMLLIQDSTDNIGQSKPLRITGPQLNVDNAVSAVHQM
Cs-Noto9    NNFNL--GG-------------QGGM-SGIEKSIEIAVPPDKCGLIIGKGGETIKMLQQSLGVKMLLIQDSTDNIGQSKPLRITGAQMNLDNAVNAVHQL
Mt-Noto9    NSGGAPLSG-------------DGQM-----ATIDVMIPPDKCGIIIGKGGETIKSLKMNLGVQMNLIQDSTMVTGQQKPLRISGDPQRVEQAKQAVEQI
Hr-Noto9    VGMPAGLEEPLM----------GAQT------VIQLMIPPDKCGLIIGKGGETIKAIQQNIGVKMMLIQDTTQNTGMDKPLRISGEPMKVEQAKQHIEQI
Sp-FUSEBP   -QNEA-----------------EGNM------TVDMLIPATKVGLVIGKGGETIKQLQEQAGVRMVMIQEGPVATGMDKPLRISGDSQKIEEAKRLVSEV
Pl-FUSEBP   -QNEA-----------------EGNV------TFDMMIPALKVGLVIGKGGETIRQLMEQAGVRMVMIQDGPGNTGADKPLRITGDASKVENAKSLVKEV
Bf-FUSEBP   TTLP------------------DGSI------VTEMMIPGNKVGLVIGKGGETIRSLQERAGVKMVMIQDGPYMNAPEKPLRITGDPQKTQRAKDLVMDL
Sk-FUSEBP   SDGGL---G-------------DGNT------TIELMIPSNKVGLVIGKGGEMIKKLQERAGVKMVMIQDATTSGTSDKPLRVSGDPQKCKHARELVNEL
Mm-FUSEBP3  FHNDM-----------------DGNS-----TIQELLIPASKVGLVIGKGGETIKQLQERTGVKMVMIQDGPLPTGADKPLRITGDPFKVQQAREMVLEI
Hs-FUSEBP3  FHNDI-----------------DSNS-----TIQEILIPASKVGLVIGRGGETIKQLQERTGVKMVMIQDGPLPTGADKPLRITGDAFKVQQAREMVLEI
Pm-FUSEBP   VDHTS--------------------H------SQEMMIPADKVGLIIGKGGETIKQLQDRAGVRMLLIQDGSQPTGADKPLRIMGEPFKVQQAKELVLEL
Mm-FUSEBP1  FHHGDG----------------PGNA------VQEIMIPASKAGLVIGKGGETIKQLQERAGVKMVMIQDGPQNTGADKPLRITGDPYKVQQAKEMVLEL
Hs-FUSEBP1  FHHGDG----------------PGNA------VQEIMIPASKAGLVIGKGGETIKQLQERAGVKMVMIQDGPQNTGADKPLRITGDPYKVQQAKEMVLEL
Mm-FUSEBP2  QFHDNANGG-------------QNGT------VQEIMIPAGKAGLVIGKGGETIKQLQERAGVKMILIQDGSQNTNVDKPLRIIGDPYKVQQACEMVMDI
Hs-FUSEBP2  QFHDNANGG-------------QNGT------VQEIMIPAGKAGLVIGKGGETIKQLQERAGVKMILIQDGSQNTNVDKPLRIIGDPYKVQQACEMVMDI

                   410       420       430       440       450       460       470       480       490       500
            ....|....|....|....|....|....|....|....|....|....|....|....|....|....|....|....|....|....|....|....|
Ci-HNRNPK   LQQAPPKGP-------IKNYDPNCFDDSY--DYGGYANERGMDGPRGG----RRG----GRGGRG-------------GFRSGGRFDRG--EKFSG----
Sp-HNRNPK   CTETAVKGP-------VQDYDPFCHDLDFYNQYGGYLFDPAEEVHGGGERGGFGG----ERGGGHWNRGPPMGRR---GRMGFDRGMSGGNDNFGGFSRS
Hs-HNRNPK   ISESPIKGR-------AQPYDPNFYDETY--DYGGFTMMFDDRRGRPV------GFPMRGRGGFDRMPPGRGG-----RPMPPSRRDYD---DMSP----
Mm-HNRNPK   ISESPIKGR-------AQPYDPNFYDETY--DYGGFTMMFDDRRGRPV------GFPMRGRGGFDRMPPGRGG-----RPMPPSRRDYD---DMSP----
Sk-HNRNPK   LNQTPIKGP-------VQLYDPYCHD--PYLDYGGYGYG-DEFGRRGG------G----GRGGRGGPRG---------GPQRFSPPMRG------G----
Bf-HNRNPK   LQKAPIKGQ-------NIPYDPFMYDEFYAAEYGGYT---SYDDQRGG------G----GRGGGDRRGGGFSGRG---GSGGGPRGRGG---PPRGH---
Od-Noto9c   LLPKEEKTA-------------------------------AKLAP---------G----GQVK---------------SEFPVPQGAVG---VIIG----
Od-Noto9a   MAQKD----------------------------GAKPAA-SIINTKTV------G----HS-----------------IEVSVPKAAVG---VVIG----
Od-Noto9b   MAAKE-----------------------------------NITHTKVP------D----GQDQ---------------VTFMIPKVSVG---VVIG----
Ci-Noto9    MANRDQQIA-------------------------------QQKMERDG------G----GGGGQGGMDPNMFGDDVNKTIIPVPKAAVG---VVIG----
Cs-Noto9    MANRDQQLA-------------------------------QQKVDRDG------S----G------MDPNMFGDDVNKTIIPVPKAAVG---VVIG----
Mt-Noto9    MQQRDEQMM--------------------RSGMGGQQTQRRFENNQPQ------G----SEN----------------VGIQVPRPAVG---VVIG----
Hr-Noto9    MAQRDLQQQ--------------QQQMQR----GGG----GGGGGNGG------G----GGRMNFPSGPQ--------KLIPVPKPAVG---VVIG----
Sp-FUSEBP   MENAKNNDR-----------------------SSG-----DNFYSRGG------P----H------------------KDVIVPKHAVG---MVIG----
Pl-FUSEBP   MENARNNQD----------------------RSGG-----DNFFSRGP------G-----------------------TEVTVPRHAVG---MVIG----
Bf-FUSEBP   ITDKELEVG----------EGEFFGGGPPGMRRGGRDFDTNDYGSARG------G----GGGG---------------MDIPVPRFAVG---IVIG----
Sk-FUSEBP   IGDKDNPGMEMFGERMDGGYDRERDDDRDFERRGGR----GDYGPRMG---GPPG----GGGG---------------FEMLVPRFAVG---IVIG----
Mm-FUSEBP3  IREKDQADF-----------------------RGVR----SDFTSRAG------G----GS-----------------IEVSVPRFVVG---IVIG----
Hs-FUSEBP3  IREKDQADF-----------------------RGVR----GDFNSRMG------G----GS-----------------IEVSVPRFAVG---IVIG----
Pm-FUSEBP   LREKDREDFR------PGNNNNNNGFGNKSGGGGGGGGGGGGGGGGGG------G----GGGGGGGGGGGGGGGGVGSMEVTVPRFAVG---IVIG----
Mm-FUSEBP1  IRDQ-----------------------------GGFREVRNEYGSRIG------G----NEG----------------IDVPIPRFAVG---IVIG----
Hs-FUSEBP1  IRDQ-----------------------------GGFREVRNEYGSRIG------G----NEG----------------IDVPIPRFAVG---IVIG----
Mm-FUSEBP2  LRERDQGGF------------------------GDR----NEYGSRVG------G----G------------------IDVPVPRHSVG---VVIG----
Hs-FUSEBP2  LRERDQGGF------------------------GDR----NEYGSRIG------G----G------------------IDVPVPRHSVG---VVIG----

                   510       520       530       540       550       560       570       580       590       600
            ....|....|....|....|....|....|....|....|....|....|....|....|....|....|....|....|....|....|....|....|
Ci-HNRNPK   ------GYGGMKRGGGRQNYGGDGGGFGG--SRY--SAPKSGRRLY--GGYDEDGYQDYD----DNFDPYQE-NLYND----S-----------------
Sp-HNRNPK   PYVGGGGGGGGGGHL--GGGGGGGGGMR---GDR--GGRGRG---R--GGR--DGGGIGG----RPRFDRDQQFGAQG----FDFGGDAG-GRGQS----
Hs-HNRNPK   ------RRGPPPPPPGRGGRGGSRARNLP--LPP--PPPPRGGDLM--AY---DRRGRPG----DRYDGMVGFSADET----WDSAIDTW-SPSE---WQ
Mm-HNRNPK   ------RRGPPPPPPGRGGRGGSRARNLP--LPP--PPPPRGGDLM--AY---DRRGRPG----DRYDGMVGFSADET----WDSAIDTW-SPSE---WQ
Sk-HNRNPK   ------QRGGMRGR---GGLGGGGGGFRG--NSR--GSPRGGNSMPFRGGRGRGGGRGGG----RGGYNYRD-DYESG----QG-----GYGGGNQGYAQ
Bf-HNRNPK   ------RGGPGGQGSRMGGNMGHRDNFHGGHGGGRAGGPRMMPSEMRSGPPPSRGLGGGGPPPMRNRGGLMSQGGQGGRGGMGGSDRMGGYGGGGRDFTR
Od-Noto9c   ------KKGETITHL--QGETVTRIQFKP--EEP--DAATRGCYIT--GSM--EGVLRAQ----QIVMSICRKKMTGV----DTIQNIPKFGQGQAPPMM
Od-Noto9a   ------RGGENISKI--QNETNTRIQFKT--DDP--TQDVRSCSIS--GTP--EACQVAN----DRISEIARQKLQEQ----H-----------------
Od-Noto9b   ------KAGETINRI--QEQTQTRIQFVP--DDP--KILERGCYII--GPQ--EGCLVAQ----KEVLEVVRKKMEEV----EGS----K-QPMP-----
Ci-Noto9    ------KGGDMINQI--QNVTGTRVQFKP--EDP--TLPERMCSVM--GPK--EGVDAAI----RRIHEIIQ-NVQER----DA-----G-NRGG-----
Cs-Noto9    ------KGGDMINQI--QNVTGTRIQFKP--EDP--GLPERMCSIM--GQK--EGVDAAI----RRIHEIIQ-NVQER----D------G-NRS------
Mt-Noto9    ------KGGDMITRI--QNETGTRIQFRE--DNP--NLPNRLCSIT--GTT--EGCRQAQ----VRVEELIQ-SVQEH----DA----------------
Hr-Noto9    ------KGGDMITRI--QNETGTRVQFKQEEEDP--NVPHRMCTIT--GPH--ENIQKAI----EKIQELIQ-SVRDH----DN-----S-GMG------
Sp-FUSEBP   ------RGGDMIKRI--QEQTKARVQFKP--GDR--DAPERVALIT--GSA--ESVSMAE----SMVNDLVA-NARDI----GMDGGDMD-GQGGP----
Pl-FUSEBP   ------RKGDMIKRI--QEQTKAKVQFTP--GDR--DAPQRVALIT--GSP--ESVSMAE----SMVSELVS-NARDM----GMD----G-SPGSEMGWM
Bf-FUSEBP   ------KGGEMIKKI--QNESGVRVQFKP--DDG--QNPNRVCQLI--GAP--DRCQAAA----HTIQNLVE-DAQQR----DQA----G-G--------
Sk-FUSEBP   ------RGGDMIKKI--QNETGARIQFRP--DDG--HSPERLAVIS--GSD--DKIDHAR----EKIDELID-SARQK----DEQRRQGG-GRDR-----
Mm-FUSEBP3  ------RNGEMIKKI--QNDAGVRIQFKP--DDG--ISPERAAQVM--GPP--DRCQHAA----RIINELIL-TAQER----EIL----G-GL-------
Hs-FUSEBP3  ------RNGEMIKKI--QNDAGVRIQFKP--DDG--ISPERAAQVM--GPP--DRCQHAA----HIISELIL-TAQER----DGF----G-GL-------
Pm-FUSEBP   ------RSGEMIKKI--QSDAGVRIQFKP--DDG--SGPERVAVLV--GSP--DCCQHAS----QLIHNLLD-SIQEG----AVP----G-GGGG-----
Mm-FUSEBP1  ------RNGEMIKKI--QNDAGVRIQFKP--DDG--TTPDRIAQIT--GPP--DRCQHAA----EIITDLLR-SVQAG----NP-----G-GPG------
Hs-FUSEBP1  ------RNGEMIKKI--QNDAGVRIQFKP--DDG--TTPERIAQIT--GPP--DRCQHAA----EIITDLLR-SVQAG----NP-----G-GPG------
Mm-FUSEBP2  ------RSGEMIKKI--QNDAGVRIQFKQ--DDG--TGPEKIAHIM--GPP--DRCEHAA----RIINDLLQ-SLRSG----PP-----G-PPGA-----
Hs-FUSEBP2  ------RSGEMIKKI--QNDAGVRIQFKQ--DDG--TGPEKIAHIM--GPP--DRCEHAA----RIINDLLQ-SLRSG----PP-----G-PPGG-----
```

```
                   610       620       630       640       650       660       670       680       690       700
             ....|....|....|....|....|....|....|....|....|....|....|....|....|....|....|....|....|....|....|....|
Ci-HNRNPK    ----------------------GDGQTG-------------NGDQK-------------------------TTQVTIPTSCAASVIGKTGQRIRQIREDS
Sp-HNRNPK    ---NFGS-----------GGGGGAGAGGF------------EGGPG----------------AGAGGEASQSQQVTIPNDLAGSIIGRGGQRIKRIRMQS
Hs-HNRNPK    MAYEPQG-----------GSGYDYSYAGG------------RGSYG-------DLGGP-----------IITTQVTIPKDLAGSIIGKGGQRIKQIRHES
Mm-HNRNPK    MAYEPQG-----------GSGYDYSYAGG------------RGSYG-------DLGGP-----------IITTQVTIPKDLAGSIIGKGGQRIKQIRHES
Sk-HNRNPK    DSMGGGG-----------YGG-GQGYDSGMDQQYN------TSQFT-------------------------TTQVTIPKDLAGSIIGKGGSRIGQIREDS
Bf-HNRNPK    DDYGGGSRFSRDRDRDGMGGGGGGGFGGGGG----------GGGFR---------SGGSDHGFGTGGN-ITSTQVTIPKELAGSIIGRGGSRIRTIRERS
Od-Noto9c    APPHNGA-----------PPMNWNQSPAG------------HGGYNGMQNSYQMPRPPPALQPEQ------TVDYPVPAQKAGAVIGKGGEHIIAIKNQS
Od-Noto9a    ------------------PPLHGNNDSSFM-----------QGQH--------------------------CVEYPVPASRAGVVIGKGGENIRLIKENS
Od-Noto9b    ---KLVF-----------DGKRYVKQDGM----N-------ASHHN----------------GGG----EQQVDYPVPASRAGVVIGKGGETINGIKEKT
Ci-Noto9     ---SGGG-----------GGGSGGGHPGG--------MGG-FGQWE----------GPN-MGRGGRGMGMASEEHLVPANKTGLVIGKGGDTIKQINMQS
Cs-Noto9     ---GGGG-----------GGGPPGGMGSF-------------GQWE----------GPN-MGRGNRGMGVSSEEHLVPANKTGLVIGKGGDTIKQINMQS
Mt-Noto9     ---SGQT-----------TRTRRPGTNS-------------FGQWE----------GPGMGGGGGGRNT--TVEFKVPANKTGLVIGKRGENIRQINQQS
Hr-Noto9     ---GGQG-----------DGSRGRNRPGG----------S-FGQWE---------GGPGGMVRGRSGPGMNTVEYNVPTNKTGLVIGKGGENIRQINQQS
Sp-FUSEBP    ---WMGS-----------GRGRGRGRGGGGPSFGSRGGGNRGGDFG---------GMGGGGGGGGGGIRGEPLDHRVPAGKCGLVIGRGGENIRAIMQQS
Pl-FUSEBP    G--GGGR-----------GGGRGRGRGGGPNFGGRGGGR--PGDFG---DFGGMGSGGGPMGGGPPGRRGEPQEHVVPATKCGLVIGRGGESIRGIMNQC
Bf-FUSEBP    ------------------GPGMGRGRG--------------RGDWG---------RGPG--GPGP----MRTDEFPVPNNKCGLVIGKGGESIRTINQQS
Sk-FUSEBP    ---GFGP-----------PPHREGGRGGG------------GGGFR---------GGP----PDR----MDSTTFTVPSSKCGLVIGKGGETIRNINMQS
Mm-FUSEBP3   ------------------TVTRGRGRG--------------RSDWS---------VGT----PGG----VQEITYTVPADKCGLVIGKGGENIKSINQQS
Hs-FUSEBP3   ------------------AAARGRGRG--------------RGDWS---------VGA----PGG----VQEITYTVPADKCGLVIGKGGENIKSINQQS
Pm-FUSEBP    ---GGGG-----------GGGRGRGRGGG----GRGGGFC-GGDWG---------SHS----PGGGGSMQEHFTYPVPTSKCGLVIGRGGETIRNISQQT
Mm-FUSEBP1   ------------------PGGRGRGRG--------------QGNWN---------MGP----PGG----LQEFNFIVPTGKTGLIIGKGGETIKSISQQS
Hs-FUSEBP1   ------------------PGGRGRGRG--------------QGNWN---------MGP----PGG----LQEFNFIVPTGKTGLIIGKGGETIKSISQQS
Mm-FUSEBP2   ---PGMP-----------PGGRGRGRG--------------QGNW-----------GP----PGG------EMTFSIPTHKCGLVIGRGGENVKAINQQT
Hs-FUSEBP2   ---PGMP-----------PGGRGRGRG--------------QGNW-----------GP----PGG------EMTFSIPTHKCGLVIGRGGENVKAINQQT

                   710       720       730       740       750       760       770       780       790       800
             ....|....|....|....|....|....|....|....|....|....|....|....|....|....|....|....|....|....|....|....|
Ci-HNRNPK    GAVIVIDEAGPGEEE---RVISITGNEEQTQNAQFLLQK-------------------------------------------------------------
Sp-HNRNPK    GAQIKIDDPLSGAKD---RIITITGTQHDIAHAKFLLQNSV-----------------------------------------------------------
Hs-HNRNPK    GASIKIDEPLEGSED---RIITITGTQDQIQNAQYLLQNSV-----------------------------------------------------------
Mm-HNRNPK    GASIKIDEPLEGSED---RIITITGTQDQIQNAQYLLQNSV-----------------------------------------------------------
Sk-HNRNPK    GALIKIDEPLPGSND---RIITIKGTPEQIQNAQYLLQNRVRAS--------------------------------------------------------
Bf-HNRNPK    EAQIKIDEPLPGSTD---RIITISGDNDQIRNAQFLLQESCSVNL-------------------------------------------------------
Od-Noto9c    GCQITQNKTNPPSNDTAWRYFTIKGQPEGVQLAQKLIQEKV-GGPPPPGANLGNAPPS-------------------------TPPHHMMQRQGYPQPPY
Od-Noto9a    GAFVQIEKNASDKGD-NWKTFIIRGTEQQIQEAQKLIQDKAGIGPPNTHTQASHSMDHFQIHGNSHTGPGTP------THQIPQTPNQGMITQLTNQVNS
Od-Noto9b    GAFVQINKN-PPAEHPDWKYFTIRGNSQQIAHAQKLIQEKV-GGPA------------------------------------------------PPGAAI
Ci-Noto9     GAHAEIQRNPPPGSDLNYKTFIIKGTPEQIKMARQLIQEKVDAGPGGSSNGQMHGGHM-----------------------GGQPPMSNFGPPPHQQGGY
Cs-Noto9     GAHAEIQRNPPPGSDLNYKTFIIKGTPEQIKMCRQLIQEKVDAGPGGQNNGQMQSSHM-----------------------GGQPPMGNFGPP-QQQGGY
Mt-Noto9     CAHVEIQRNPPPGSDPQFKTFILKGTTEQIQYAQQLIQERVDGGPGGAATGGAATQSY------------------------GAPPMQSGYQAAPQGGYA
Hr-Noto9     QAHVEIQRNPPPGSDQMYKTFIIKGTPEQIALAQQLIQEKVEGGPGGGGNH----------------GHGXPGHVGGMGIMGHGPPGAHHGPGPGGPGGY
Sp-FUSEBP    RAHVEISHGQHP---PGQKIFLISGDPEQIDYARSLIDEKVNGSPGGGPGGPG----------------------------GPGGPGGPGGP--GGGGPG
Pl-FUSEBP    GAHVEISHGQHP---PGQKVFLITGEPDQVEYARSLIDEKVKGEPGGGPRG-------------------------------PGGPGGAGGPGGPGGGPG
Bf-FUSEBP    GAHVELMRNPPPHCEPGMKMFSIRGSPQQIDHAKQLIHEKISDDMHEPFGFGDR----------------------------PFHPRKHGGPPGPPGGPG
Sk-FUSEBP    GAHVELSRNLGP---PGEKVFTIRGSPQQISCAQQLIHEKVSGGPGGPGGPGGPGGPG---------GPGGPGPGGPHGGHGHGGP---GGPPGGQGGPG
Mm-FUSEBP3   GAHVELQRNPPPNTDPNLRIFTIRGAPQQMEVARHLIDEKV-GG--ASLGAPAAFGQS-------------------PFSQPPAAPHQNTFP--PRG---
Hs-FUSEBP3   GAHVELQRNPPPNSDPNLRRFTIRGVPQQIEVARQLIDEKV-GG--TNLGAPGAFGQS-------------------PFSQPPAPPHQNTFP--PRSSGC
Pm-FUSEBP    GAQIEIQRNAPPGADPNFKLFFIRGGQQQIEHARQQIDNIVNSG---------------------------------------------GPPGPPCGFPG
Mm-FUSEBP1   GARIELQRSPPPNADPNMKLFTIRGTPQQIDYARQLIEEKI-GGPVNPLGPPVPHGPH-------------------GVPGPHGPP---GPP--GPGTPM
Hs-FUSEBP1   GARIELQRNPPPNADPNMKLFTIRGTPQQIDYARQLIEEKI-GGPVNPLGPPVPHGPH-------------------GVPGPHGPP---GPP--GPGTPM
Mm-FUSEBP2   GAFVEISRQLPPNGDPNFKLFVIRGSPQQIDHAKQLIEEKI-EGPLCPVGP---------------------------------GP---GGP--GPAGPM
Hs-FUSEBP2   GAFVEISRQLPPNGDPNFKLFIIRGSPQQIDHAKQLIEEKI-EGPLCPVGP---------------------------------GP---GGP--GPAGPM

                   810       820       830       840       850       860       870       880       890       900
             ....|....|....|....|....|....|....|....|....|....|....|....|....|....|....|....|....|....|....|....|
Ci-HNRNPK    ----------------------------------------------------------------------------------------------------
Sp-HNRNPK    ----------------------------------------------------KEYQNSGGALQ-------------------------------------
Hs-HNRNPK    ----------------------------------------------------------------------------------------------------
Mm-HNRNPK    -------------------------------------------KQYSGKFF-------------------------------------------------
Sk-HNRNPK    ----------------------------------------------------QSYQQQSFGQDM------------------------------------
Bf-HNRNPK    -----------------------------------------------GRTFAVRYG--------------------------------------------
Od-Noto9c    GAPPQRMPPHMPQPP-----QTGPPQFQSPGMPPVVQ------HGAQHQQY-QHPSTPQYPPQGPPAAPAPVQPAPVAQPQQSTDYHAAWAAYYQQM---
Od-Noto9a    LNLHPN--PGTPGPP-----PPSNQPGIGGPWPQVDQHPDSVNQGW--NQYPMYGGQPANGWQPWNTGNNPHQ---QPRSQQQQNLSSQWAQYYEHA---
Od-Noto9b    ANNSSG--NGYSYTG--------------------------------GNSYGSNNSGGGGQQD----------------------YSAAWAQYYASI---
Ci-Noto9     GGLPPA--AGYGHPS----HNPLQGPPQAPQAPQSYQ------QPW-GNQF-QQWNSPTVSAP-QQNGADPTK------PVADSSNAAAWQTYLQMY---
Cs-Noto9     GGLPPAASYGHPNPL--------QGPPQAPQAPQSYQ------QPW-GSQF-QQWSSPSVSAP-QQNGADPTK------PVADSSNAAAWQSYLQMY---
Mt-Noto9     QPAAPQQAQGYGSQP-----------------------------AWNQQPY-QQWTQPP-----------------------------------------
Hr-Noto9     GPPPPQNNFGAPPPGPGGFGGPPQGPPQQAQPPQQFQNQ----QPWAGAPNQYQWPQPGGPQQNGAIPQDPNK----PPDS----NAAAWANYYNYYQNQ
Sp-FUSEBP    GPQGHR--GGFGGPP--------GSGPQQAPVQQGYNNQ----QQW--NAY-SQWQQPQ-QPQQQPPQQQPHQ---PAPDASAYGDSNPYAAYYQQY---
Pl-FUSEBP    GPQGHR--GGFGGPP---------GGPQQNPVQPGYNNQ----QGW--NAY-SQWQQPQQPQQ-QPPQQQPHQ---PAPDTSGYGDSNPYAAYYQYYQQA
Bf-FUSEBP    GPG-----GFNQGPP-----PPNQPPPPGGGPPPYAP------QGW-GNAYQQQWQQGQGGPN------DPNK--------AAQDNAAAWQAYYAQY---
Sk-FUSEBP    GPGGPG--GFNQAPP------QGGPGGQGGPPPPQAFTP----QGW-GNAY-QQWQQHN--PQ------DPSK---AAADA----NAAAWAAYYQQY---
Mm-FUSEBP3   FPNIAA--KVNGNPH--------STPVSG---PPA-FLT----QGW-GSTY-QAWQQPT--QQ------VPSQ---------------------------
Hs-FUSEBP3   FPNMAA--KVNGNPH--------STPVSG---PPA-FLT----QGW-GSTY-QAWQQPT--QQ------VPSQ---------------------------
Pm-FUSEBP    QGQGPG--PFHPGPP---------GPPRGPPSQPFEQ------PGW--GNFQQPWQQPQQPPM--PPAMNPGN-------APADPNAANWAAYYGHQ---
Mm-FUSEBP1   GPYNPA--PYNPGPP--------GPAPHG---PPAPYAP----QGW-GNAY-PHWQQQA--PP------DPAK-------AGADPNSAAWAAYYAHY---
Hs-FUSEBP1   GPYNPA--PYNPGPP--------GPAPHG---PPAPYAP----QGW-GNAY-PHWQQQA--PP------DPAK-------AGTDPNSAAWAAYYAHY---
Mm-FUSEBP2   GPFNPG--PFNQGPP--------GAPPHAGGPPPHQYPP----QGW-GNTY-PQWQPPA--PH------DPNK----AAAAATDPN-AAWAAYYSHY---
Hs-FUSEBP2   GPFNPG--PFNQGPP--------GAPPHAGGPPPHQYPP----QGW-GNTY-PQWQPPA--PH------DPSK----AAAAAADPN-AAWAAYYSHY---
```

```
                     910       920       930       940       950       960       970       980       990      1000
            ....|....|....|....|....|....|....|....|....|....|....|....|....|....|....|....|....|....|....|....|
Ci-HNRNPK   ----------------------------------------------------------------------------------------------------
Sp-HNRNPK   ----------------------------------------------------------------------------------------------------
Hs-HNRNPK   ----------------------------------------------------------------------------------------------------
Mm-HNRNPK   ----------------------------------------------------------------------------------------------------
Sk-HNRNPK   ----------------------------------------------------------------------------------------------------
Bf-HNRNPK   ----------------------------------------------------------------------------------------------------
Od-Noto9c   ---NSQPQNPAPATPTPQPAAA------------------------------------------------------------------------------
Od-Noto9a   KIQDHHAHSYQQPHMIHQQSSQPNISSADPG-------------------------------QWSDPYRGHLGTGYHP--G-------------------
Od-Noto9b   SQQQAAS----------APAAAPA--------------------------------------------------------A-------------------
Ci-Noto9    ---GAQAQAYQQPAAAAAVSAA----------------------------------------------------------P-------------------
Cs-Noto9    --GAQAQAAYQQPTAAAAVSAAP---------------------------------------------------------P-------------------
Mt-Noto9    -QQNGTTADQMPGGMPQMAGGA----------------------------------------------------------A-------------------
Hr-Noto9    GQQPAAAAAAVAAAYPQQAAAAPGTVVHTQT-------------------------------------------------A-------------------
Sp-FUSEBP   YQQQQQQNPTSQTASTVQTTPSTSAPQSTDAQQGQANN------------------------------------------G-------------------
Pl-FUSEBP   AQQQQQQAATSQPASTVQTTPSSSAPQST-----------------------------------------------------------------------
Bf-FUSEBP   YNWQQQAQPPAPGQGPPQQAQQPGGGSGQQPDYSKAWEEYWKRTGEQPQQPQQQQQPQQPGGQQGAPAQQQTQQAGQQAGQ-------------------
Sk-FUSEBP   YQQQNQPNSQQQNQPSNQQSQQQQSPQQQQPPQSSTSSSTTAPGTGGSGAGGGGSSGSGSNSQSQNPGS------QAPK-G-------------------
Mm-FUSEBP3  ---QSQP-------------------------------------------------------------------------Q-------------------
Hs-FUSEBP3  ---QSQP-------------------------------------------------------------------------Q-------------------
Pm-FUSEBP   ---GAMPGGMPGGMPPGMPGGMPAGMPGAMPGGMPGGMPGGMPGGMPGGMPGGMPGGMPGGMPAGMPGGMPAGMPGAMPGGMQGGMPGNMQAAMQTAMQP
Mm-FUSEBP1  YQQQAQP-------PPAAPAGAPA--------------------------------------TTQTNGQGNYGDQQAPA-P-------------------
Hs-FUSEBP1  YQQQAQP-------PPAAPAGAPT--------------------------------------TTQTNGQ---GDQQNPA-P-------------------
Mm-FUSEBP2  YQ---QP-------PGPVPGPAPA--------------------------------------PAAPPAQ---GEPPQPP-P-------------------
Hs-FUSEBP2  YQ---QP-------PGPVPGPAPA--------------------------------------PAAPPAQ---GEPPQPP-P-------------------

                    1010      1020      1030      1040      1050      1060      1070      1080      1090      1100
            ....|....|....|....|....|....|....|....|....|....|....|....|....|....|....|....|....|....|....|....|
Ci-HNRNPK   ----------------------------------------------------------------------------------------------------
Sp-HNRNPK   ----------------------------------------------------------------------------------------------------
Hs-HNRNPK   ----------------------------------------------------------------------------------------------------
Mm-HNRNPK   ----------------------------------------------------------------------------------------------------
Sk-HNRNPK   ----------------------------------------------------------------------------------------------------
Bf-HNRNPK   ----------------------------------------------------------------------------------------------------
Od-Noto9c   ---------------------------------------------------------------AAQPDYTKAWEEYFKVQKHQQSNNGSAAQ--------
Od-Noto9a   ---------------------------------------------------------------QGYPGNTNQPNFHYQDLGLSSL---------------
Od-Noto9b   ---------------------------------------------------------------TGQPDYSKAWEEYFKKTGQTPQSYQAS----------
Ci-Noto9    ---------------------------------------------------------------ASQPDYSKQWDEYLKQNAAAVAEQRQQLTLAAPAAAA
Cs-Noto9    ---------------------------------------------------------------ASQPDYSKQWDEYLKQNGLTAAAVAERQQLALASPAA
Mt-Noto9    ---------------------------------------------------------------PAVPDYSKQWEEYFKKQGEQTGAAAPQGQ--------
Hr-Noto9    ---------------------------------------------------------------AGQPDYTKAWEEYYKKQAEAAAPAA------------
Sp-FUSEBP   ---------------------------------------------------------------AGNRDMLRQWAEYYKNQA-------------------
Pl-FUSEBP   ---------------------------------------------------------------DAQPGQGAA----------------------------
Bf-FUSEBP   ---------------------------------------------------------------SGQPDYTKAWEEYYKRQAQSQQ---------------
Sk-FUSEBP   ---------------------------------------------------------------DGQPDYSAAWAEYYRQQAQAQ----------------
Mm-FUSEBP3  ---------------------------------------------------------------NSQPDYSKAWEDYYKKQSHPTS---------------
Hs-FUSEBP3  ---------------------------------------------------------------SSQPNYSKAWEDYYKKQSHAAS---------------
Pm-FUSEBP   GVQPGMQAQQMGMMGAQQPQQQQQQQQQQQQQQQQQQQQQQQQQQQQVAAQQAADPNQATVGQNGQVDYTKAWEEYYKSIGQQ-----------------
Mm-FUSEBP1  ---------------------------------------------------------------AGQVDYTKAWEEYYKKMGQAVPAP-------------
Hs-FUSEBP1  ---------------------------------------------------------------AGQVDYTKAWEEYYKKMGQAVPAP-------------
Mm-FUSEBP2  ---------------------------------------------------------------TGQSDYTKAWEEYYKKIGQQPQQPGAPPQQDYTKAWE
Hs-FUSEBP2  ---------------------------------------------------------------TGQSDYTKAWEEYYKKIGQQPQQPGAPPQQDYTKAWE

                    1110      1120      1130      1140      1150      1160      1170      1180      1190      1200
            ....|....|....|....|....|....|....|....|....|....|....|....|....|....|....|....|....|....|....|....|
Ci-HNRNPK   --------------NAEEPKDE------------------------------------------------------------------------------
Sp-HNRNPK   ----------------------------------------------------------------------------------------------------
Hs-HNRNPK   -------------------------------------------KQYADVEGF------------------------------------------------
Mm-HNRNPK   ----------------------------------------------------------------------------------------------------
Sk-HNRNPK   ----------------------------------------------------------------------------------------------------
Bf-HNRNPK   --------------------------------FELVLHFVAQY---------------------------------------------------------
Od-Noto9c   --------------PTQATGSQ----------PDYTAAWAEYYKSMG-YYGKS-----------------------------------------------
Od-Noto9a   --------------LEDSLEAK----------LNLSLCSDSEFDPFEPYEPYDPALDSSRFTPQSQC---------------------------------
Od-Noto9b   -----LAAQQPAAAPAPTPAAA---P--ATGGQDYSAQWAEYYRKLAEYQKAQGN---------------------------------------------
Ci-Noto9    AIQQQQVAAPAAAAPAASAAVQ----PATTQAADYSAAWAEYYRQYYAMHGQAGYAAAAAAAGQAPAPT-------------------------------
Cs-Noto9    AAAIQQQVAAPAAAAAAPTAAAAAVAQPATTQADYSAAWAEYYRQYYAMHGQAGYAAAAAAAGQAPAPT-------------------------------
Mt-Noto9    --------------APPTAGTPQPATSQAAAGVDYSQAWADYYRQYSQYYQQQTQYSAGQQPAQQ-----------------------------------
Hr-Noto9    --------------VNPAAASQ-------AGGADYSAAWAEYYRQYAQYYQQSQAQQQYSTAPAAPAQQ-------------------------------
Sp-FUSEBP   --------------AGQAGGQP----------QDYSQQWMEYFQQQALYFNQPNQGQTPAQGQ-------------------------------------
Pl-FUSEBP   --------------TGQAGQPQ----------QDFSQQWMEYFQQQAMYYNQPNQGQTPAQGQ-------------------------------------
Bf-FUSEBP   --------------QQQAAQPQ----------QDYSQAWADYYRQYGGAPGQSQPGQQLLQAENSQEDDQVGV---------------------------
Sk-FUSEBP   --------------PQPGAGNQ----------PDYSNAWAEYYRQQGMYYGQAGGQQSNQPTNQPPSQQGQ-----------------------------
Mm-FUSEBP3  --------------AAPQASSP----------PDYTMAWAEYYRQQAAFYGQTLGQAQAHSQVCSQSPAP------------------------------
Hs-FUSEBP3  --------------AAPQASSP----------PDYTMAWAEYYRQQVAFYGQTLGQAQAHSQEQ------------------------------------
Pm-FUSEBP   --------------PGQPAGAQ----------PDYTKAWEAYYKSQASAQAAVTGGSD------------------------------------------
Mm-FUSEBP1  --------------AGAPPGGQ----------PDYSAAWAEYYRQQAAYYAQTSPQGMPQHPPAPQGQ--------------------------------
Hs-FUSEBP1  --------------TGAPPGGQ----------PDYSAAWAEYYRQQAAYYAQTSPQGMPQHPPAPQGQ--------------------------------
Mm-FUSEBP2  EYYKKQAQVATGGGPGAPPGSQ----------PDYSAAWAEYYRQQAAYYGQTPGPGGPQPPPTQQGQQQASGNCHPPPPPFSFQPPATVHPALVGSAGN
Hs-FUSEBP2  EYYKKQAQVATGGGPGAPPGSQ----------PDYSAAWAEYYRQQAAYYGQTPGPGGPQPPPTQQGQQQAQ----------------------------
```

```
              ....|...
Ci-HNRNPK     --------
Sp-HNRNPK     --------
Hs-HNRNPK     --------
Mm-HNRNPK     --------
Sk-HNRNPK     --------
Bf-HNRNPK     --------
Od-Noto9c     --------
Od-Noto9a     --------
Od-Noto9b     --------
Ci-Noto9      --------
Cs-Noto9      --------
Mt-Noto9      --------
Hr-Noto9      --------
Sp-FUSEBP     --------
Pl-FUSEBP     --------
Bf-FUSEBP     --------
Sk-FUSEBP     --------
Mm-FUSEBP3    --------
Hs-FUSEBP3    --------
Pm-FUSEBP     --------
Mm-FUSEBP1    --------
Hs-FUSEBP1    --------
Mm-FUSEBP2    PFPCGVCP
Hs-FUSEBP2    --------
```

Calumenin Multiple Alignment

```
                              10        20        30        40        50        60        70        80        90       100
                     ....|....|....|....|....|....|....|....|....|....|....|....|....|....|....|....|....|....|....|....|
Ci-Reticulocalbin-1  -MRLYLVCVLLLVSCGLLGVIGRSVI-------------------KKEGQP-----KKQSTLDKEVLFEDEDYEDDDVLDLPPEEQKSRLKSLVEKKMDVN
Cs-Reticulocalbin    ----MMKLSSLLVFTTT--IFHCK--------------------AQELHT------GGGKSDKEVLFGLEEGQD-DIFNLPESEQKELLAKLVQNKIDQG
Ci-Reticulocalbin-2  MERINLNCFVLLMILVFRATWGKEMA------------------------------TGGGKSDKEVLFGGEDDDVFN---LPEATQKELLLKLVKNKMDLN
Od-Calumenin2        ----MTRIAFLLPIPFFARGTSHP-------KNPTI-DNP--------------VDSFNSHVDGILDQNESLKFH--KLTKEEKLASLEEIFK-KMDAD
Sp-Calumenin2        MMKSVLFAYVVAVVISL--AVCKPND--HEGSSRVKQETK----LSDQAHFDEHGKHNPDYDHDAFLGEEEAKKFT--NLSPEESKEKLGQLFD-RVDLN
Od-Calumenin1        ---MKFSALSIALSVNAG-RIAEPQKSKLDEKKEMLQDIKDRPLVPDMDR------DDPDYDPAALW-QDEFKTFD--DLTHEEARDKLILLIE-KMDRD
Cs-Calumenin1a       ----MLREVLFLYFVYV--AFALEH------KDRVI-DSK----LSDKEF------GTADYDHDAFLGKETAAEMD--ELSPEESKRRLAIIIT-KVDKN
Ci-Calumenin1a       ----MWRKLVLLLFLGV--GLAAV-------KERVI-DSK----LSDKEF------GTADYDHEAFLGKETAQELD--DLAPEESKRRLAIIVK-KVDKN
Ci-Calumenin2        ---MLIRCVVILLVASA--AFAGVAR-----QDRVL-DPK----LSDKEP------GSPEYDHEAFLGKDQAEELD--ELPEEESKRRLGIIVD-QVDKN
Cs-Calumenin2        -MVVVGKLYFLLLSVSV--AIAAVAK-----KDRIL-DQT----LSDKEP------GSPDYDHEAFLGKDQAQELD--ELPEEESKRRLGIIVD-KVDKD
Cs-Calumenin1b       ---MILKVFLLLFVCYV--GFAAVAK-----KDRVL-DQK----LSDKEP------GSPDYDHEAFLGKDQAQELD--ELPEEESKRRLGIIVD-QVDKD
Ci-Calumenin1b       -----MKGLMVLLMCTV--AFAAVAK-----KERVL-DQK----LSDKEP------GSPDYDHEAFLGKDQAQEHD--ELPEEESKRRLGIIVD-QVDKD
Sp-Calumenin1        MKTSLIVCMYLALLIAV--TLAKPAD-----KGRVKEEVK----LSDEEHFEGN-EHNPEYDHDAFLGHEDAKTFD--NLSPEESRERLGKIVE-KIDAD
Pm-Calumenin         -MACGWQKVAFVALCAWL-ALAKPTA---EKKNRVHPDEP----LSEKKH--DD-EQSFDYDHEAFLGAETAKTFD--QLSPEESKERLGKIVE-KIDSD
Sk-Calumenin         -MKSTSSVLSIALGILFLSVVAKPTDKNQARKDRIL-EEK----LSDKDHYDGD-LHNPDYDHEAFLGQDGASEFD--ELSPEESKTRLGQIYD-KIDKD
Bf-Calumenin         -MQNLLLLLVVLTTLTF--AIPKPTD---GKKDRVV-DKP----LSEQDHY-GD-NHNSDYDHEAFLGED-AHTFD--QLDPEESKKRLGDMVD-KIDKD

                             110       120       130       140       150       160       170       180       190       200
                     ....|....|....|....|....|....|....|....|....|....|....|....|....|....|....|....|....|....|....|
Ci-Reticulocalbin-1  QDGFIDAKELHSWTLKAFDSFENDDAKEEFSM-VDVDKDGAVSWREHSDDAHGKGYGEESPEFANPEAEEGIEKKETYLKDKKIFAAADRDGNEILDLME
Cs-Reticulocalbin    KDGFVDYAELESWSLKSLNDFETEDSRQEFSS-VDENEDGKITRKEYSSFLYGEDFDIESEQF-QGLSPDWKNFLKTYNKDTAMFTAADRDNDGSLVLDE
Ci-Reticulocalbin-2  KDEFVDREELVQWSQKSLNRFETEASREEFSS-VDENEDGKVTWEEYSSFLYGEDFAIDHEDFKNPQSDEWSGFVDRYNREKVMFAAADDNTDHGLTLDE
Od-Calumenin2        ENGEVDEDELQQWMRYVENRFVFEDTDEKLAQ-MDLDKNGMVSIREFNEAKYN------PERIYQDPSMNAATAMYQKKKDIRRFNAADINDDQHLSRNE
Sp-Calumenin2        KNGSISESELSAWIEIQTNSVLYGELDRLFKA-HNMNGDDLLTWAEYNHTTYS---GLPLEKLITMQEDKTLDFRKKVRQDKARWSLADQNRDDALDREE
Od-Calumenin1        KDGKVTEEELTIWIHYVQTKYIYDDTERQWEE-NDTDKNGKITWEEYSKHTYG---FLTDDQL-NEEEEDGFSYKAMLERDERRWKASDRENKGYLTKED
Cs-Calumenin1a       GDGIITEKEMKDWIRFTHQRYIREDSDKRFMELMQNNGTGMLHWNAFKHMVYG---YGEDGQI-VDEIHETEEYRKQFLRDERRWKRADLDGDNQCTIEE
Ci-Calumenin1a       EDGSVTEQELEDWVRLTHNKYISEDSDKRFRQLVEENQGEPLHWNNYKKMVYG---YGENGEL-VHEVDETEDYRKMYEREEKRWKRADSDEDGVLTLEE
Ci-Calumenin2        RNGQVTETELLEWIKFTQKRYVDEDAEKQFKI-YDKNNDNMVHWDEYKVTTFG---FLEDDQE-QVNGEDSESYRKMTERDHRRFREADVDKDDRCTKEE
Cs-Calumenin2        GDGKVSETELVDWIKFIQKRYINEETDKQYKL-FDKNEDGKIHWNEYKENTYG---LVEEDEY-PGSDETKESFKRTIERDQRRFKQADADKDDHCTKEE
Cs-Calumenin1b       GDGNVTETELIEWIKFTQKRYIDEDSNKQFLQ-HDTNKDGKIDWEEYKESTYG---FMKDHE--NSDDEDGLSYKKMIDRDQSRFNAADSDQNKECTPDE
Ci-Calumenin1b       GDQNITEQELVDWIKFTQKRYIREDSNKQFLQ-HDTNKDGKIEWDEYKEATYG---FMEEQA--NQDEEDGFSYKKMIDRDHKRFQAADGDKNGNCNEEE
Sp-Calumenin1        KDGFVTEEELKDWILLQQSRYIYEDVDRQWKG-HNVDGDPKITWQEYNQTTYS---GLTEEELSRMQENQHMDFSTMIRRDKKRWKVADMDNDGDLTYEE
Pm-Calumenin         GDGLVTQDEMTMWIKHTQRRWVYEDVDRQWTG-HDIDGDGSVSWEEYRNATYG---FLAEEA--QGGEEDGFNYKQMLGRDERRFKAADVDGDGQASKEE
Sk-Calumenin         NDGFVTDEELKDWIKYTQNRYIMEDVDRQWNV-HDTDKNGHLTWDEFKNTTYG---YLADDDF---DDIEGFDYKDMIRRDERRWQRADTDGDGKLSKEE
Bf-Calumenin         QDGFVTQQELKEWIQYTQKKYILEDVERQWSS-HDLNRDDKIHWDEYKNTTYG---FMSPEEELEDDEDDGYNIKDMVKRDLRRWETADSDDDKHLTKEE

                             210       220       230       240       250       260       270       280       290       300
                     ....|....|....|....|....|....|....|....|....|....|....|....|....|....|....|....|....|....|....|
Ci-Reticulocalbin-1  YFNFKHPRRNPETSQVLIEDKLESLDANKNGGIDLEEYLKDT-----------KNADEDE----------------ALAESETERFGEL-DGDGDGVLRG
Cs-Reticulocalbin    YINFKHPQFSPATREVLLADKLSSADLNKDGGIDLDEFLTDS-----------KEHDTHK----DP----------DWKIVETDKFTEDLDQNKDGVLKG
Ci-Reticulocalbin-2  YIDFKHPQFNPKTKRLLLNETLSRVDLNMDGGISLEEFLAD------------YKKNNKEN---DQ----------DWKIVETDKFKEDLDLDKDGLLKG
Od-Calumenin2        FAHYLHPTGHDEMMEVIALETLEDLDRNNDGFIDVNEYLGDIGAIEKQESVEKIVPMDDDEFENDPNIWEAPGIDEDWIENERRIFNEERDIDGDGFLNM
Sp-Calumenin2        YMAFEWPREKIHMKDVAIAETIEDIDTDGDGYVNFDEFMKDL-----------WDGQGE-----MP----------DWVEAERKGFAEYRDKDGDGKLNH
Od-Calumenin1        LTAFLHPEEYDHMKELVILETIEDIDKDGDGKIGLSEYIGDM-----------WIEEEDGA---EP----------EWVEEERKQFQMFRDKDNSGFLED
Cs-Calumenin1a       FRIFSHPEEFRNMSDLVVQETIEDMDKNDDGSIDLEEYIKDM-----------FNPENGEP---EP----------DWVRNEREHF-NIRDTNSNGKMDA
Ci-Calumenin1a       FRGFSHPEEYPHLHDIVVSETMEELDKDNDGGIDLKEYVSDV-----------YHPNNEEP---EP----------DWVQNEREQF-EARDVNKNGKMDA
Ci-Calumenin2        FKAFLHPEEFEHMRDLVARETLEDIDKNKDGFVDVKEYIGDM-----------RRDDDEKE---NL----------EWVVHEEEQFKDIRDTNGDGKMDV
Cs-Calumenin2        FKAFLHPEEYDHMKEVVAMETLEDIDKNKDGFVDLQEYIGDM-----------TSESENTGE--KP----------DWVVKEEEQFREHRDKNKDGKLDL
Cs-Calumenin1b       FKAFLHPEEFDHMKDIVTRETLDDIDKNKDGFVDLNEYVGDM-----------ISDEEKEGE--KP----------DWVVNEEEQFREHRDKNKDGKLDL
Ci-Calumenin1b       FRAFLHPEEYDHMKEIVTKETMDDIDKNSDGFVDLKEYIGDM-----------ISEDESEGE--KP----------DWVVVEEQQFHEHRDKDKDGKLNL
Sp-Calumenin1        FVGFLHPEEKGHMREIVVEETMEDIDQNGDGFVDIDEYIGDM-----------WPKSEREKGGAEP----------DWVQTEREQFFAFRDRDGDRKMDR
Pm-Calumenin         FTAFLHPEEFEHMKDIIVTETMEDIDKNGDGFIDLNEYIGDM-----------YNQAGESE---EP----------EWVKTERQQFTDFRDTNKDGKMDK
Sk-Calumenin         FAHFLHPEEGEHMRDIVVEETMEDIDKDGDGMISLEEYIGDM-----------YPSDDDED---EP----------DWVKIEREQFTRFRDKDKDGKMNK
Bf-Calumenin         FQSFLHPEDVEHMKDIVVQETLEDIDKDGDGTISLEEYIGDM-----------WTGDDKE----EP----------DWVKSEREQFGTFRDKNGDGKMDK

                             310       320       330       340       350       360
                     ....|....|....|....|....|....|....|....|....|....|....|....|....|
Ci-Reticulocalbin-1  SELLQWIDPDNSEEADDEADHLMTESDKDEDGKLSPDEIVNNHELWVESDATDYGRQLMLNHDEF
Cs-Reticulocalbin    EEIVQWVASDNTHEARDEADHLIEESDQDGDGKLSVDEILNNHALWVDSDATDYGRQLLKNHDEL
Ci-Reticulocalbin-2  EEVVMMVATDNFKEAEDEADHLIEETDEDQDGKLSPDEIVNNHELWVESDATDYGRQLMLNHDEL
Od-Calumenin2        AEVMLWMNPADFDVIQSEADFLIATADSDISSTLSKEEVLKAYDAFIESPATHWG-GSLLHHDEL
Sp-Calumenin2        EEVGDWIMPTHYDPIESEAKHLMYETDENKDNELTKEEMILHFKLFVGSRVTNFG-EL--RHDEF
Od-Calumenin1        DEVRDWILPSEYDHAEGEARHLIESADSDKSGFLSKDEILEHQDVFVGSQATDWG-DAIVRHTEF
Cs-Calumenin1a       EEVMKWILPEDYDHAESQAMHLIFEADDDKNGELTNDEIMNHHSAFVGSQLTDWG-EALKRHDEF
Ci-Calumenin1a       DEVKEWILPTDYDHAKSEARHLVHEADDDKDGELSTEEILLHHATFVGSQVTNYG-EAL-KHDEF
Ci-Calumenin2        TEIKDWILPADYDHASAEAKHLVYTADDDKDGELSKEEILNHHDTFVGSQATDWG-EALKRHQEF
Cs-Calumenin2        DEVRDWIMPNDYDHAASEAKHLIYSSDDNEDGELTKEEIIAHHDMFVGSQATDWG-EALKRHQEF
Cs-Calumenin1b       DEVRDWIMPNDYDHAASEAKHLIYNSDDNKDGGLTKEEIIAHHYVVIGSQATDWG-EALKRHDEF
Ci-Calumenin1b       EEVRDWIMPSDYDHAVAEAKHLVHESDDDKDGSLSKEEILAHHDKFVGSQATDWG-EALKRHDEF
Sp-Calumenin1        EEIGQWILPEDYDHAQAEAQHLLMESDTDNDKKLTKAEILDKYDLFVGSQATDFG-EALTRHDEF
Pm-Calumenin         EETMAWILPSDYDHAEAEARHLIYESDSDKDGKLSKEEILDKHDLFVGSQATNFG-EALTRHDEF
Sk-Calumenin         REVKDWIMPEDYDHAEAESKHLVYESDVDKDGKLSKKEVLDKHDLFVGSQATDFG-EALIRHDEF
Bf-Calumenin         DEVRDWIIPPDYDHADAESKHLIFESDVDKDQKLTKQEIVDKYDLFVGSQATDFG-EALVRHDEF
```
