# Supplementary figures and images for: Evolutionary changes in the notochord genetic toolkit: a comparative analysis of notochord genes in the ascidian Ciona and the larvacean Oikopleura
Source: BMC Evol Biol. 2011 Jan 20;11:21. doi: 10.1186/1471-2148-11-21 (PMC3034685; doi:10.1186/1471-2148-11-21)

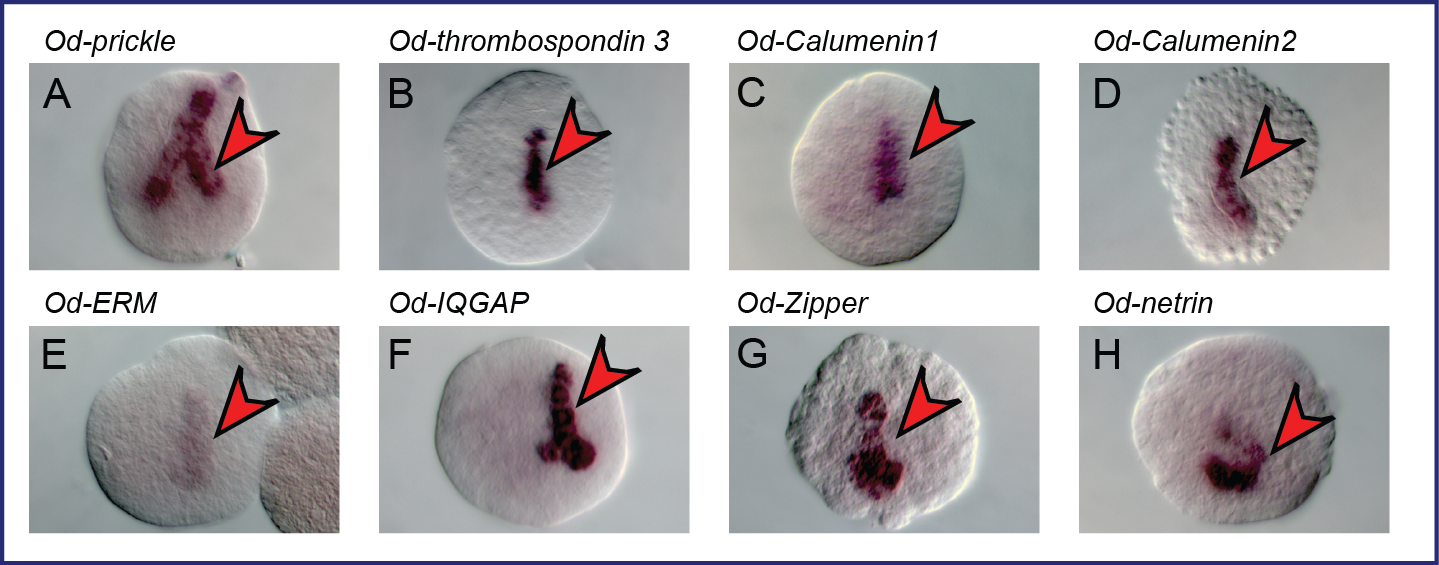

Supplement: Additional file 5 — Figure S2. Whole-mount in situ hybridization of Oikopleura neurulae. [file 1471-2148-11-21-S5.PNG]

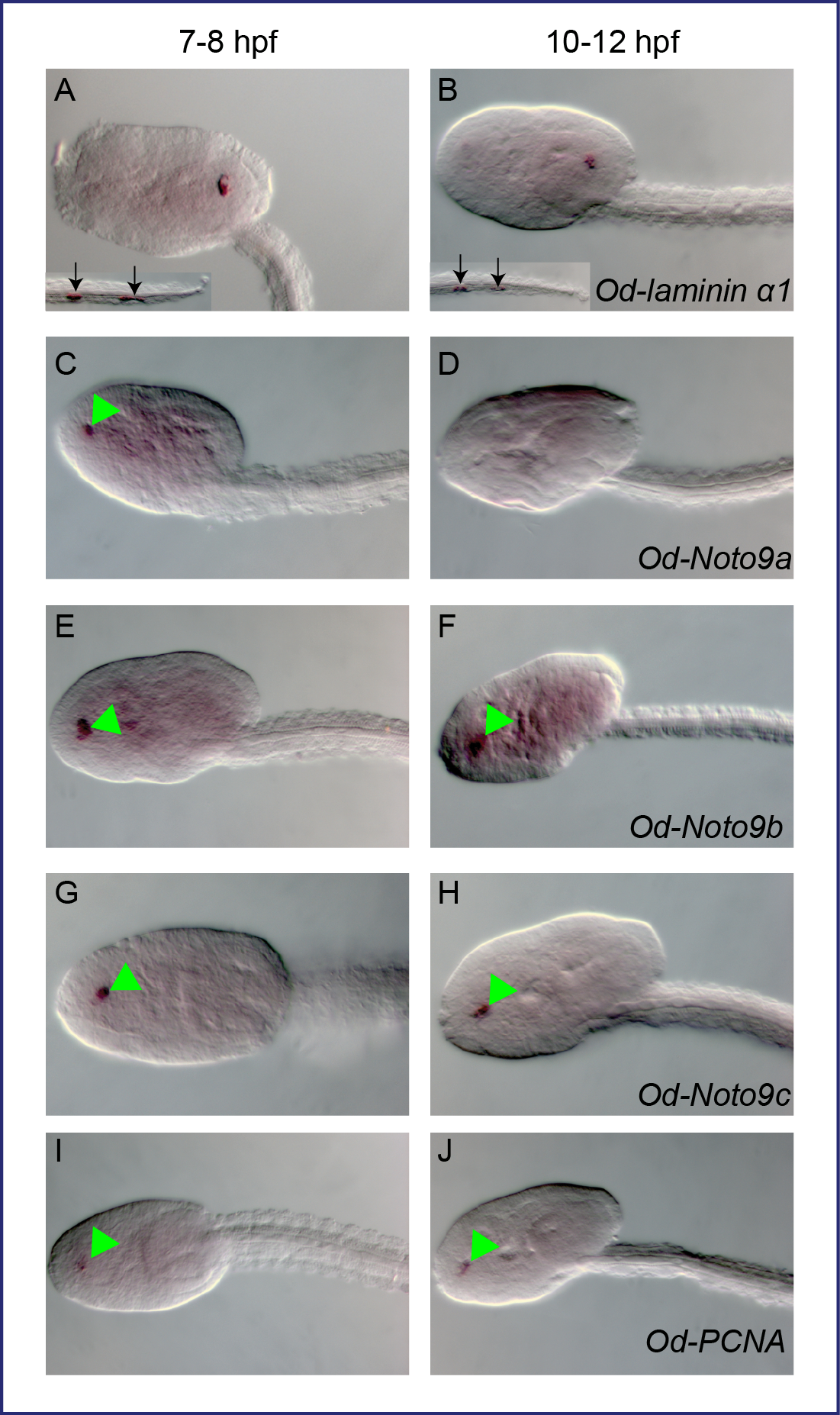

Supplement: Additional file 6 — Figure S3. Additional expression patterns in Oikopleura embryos at 7-8 and 10-12 hpf. [file 1471-2148-11-21-S6.PNG]
